# Loss of cardiomyocyte CYB5R3 impairs redox equilibrium and causes sudden cardiac death

**DOI:** 10.1172/JCI147120

**Published:** 2022-09-15

**Authors:** Nolan T. Carew, Heidi M. Schmidt, Shuai Yuan, Joseph C. Galley, Robert Hall, Helene M. Altmann, Scott A. Hahn, Megan P. Miller, Katherine C. Wood, Bethann Gabris, Margaret C. Stapleton, Sean Hartwick, Marco Fazzari, Yijen L. Wu, Mohamed Trebak, Brett A. Kaufman, Charles F. McTiernan, Francisco J. Schopfer, Placido Navas, Patrick H. Thibodeau, Dennis M. McNamara, Guy Salama, Adam C. Straub

**Affiliations:** 1 Heart, Lung, Blood and Vascular Medicine Institute,; 2 Department of Pharmacology and Chemical Biology,; 3 Division of Pulmonary, Allergy and Critical Care Medicine, Department of Medicine, and; 4 Division of Cardiology, Department of Medicine, University of Pittsburgh, Pittsburgh, Pennsylvania, USA.; 5 Department of Developmental Biology and Rangos Research Center Animal Imaging Core, Children’s Hospital of Pittsburgh, University of Pittsburgh School of Medicine, Pittsburgh, Pennsylvania, USA.; 6 Andalusian Center for Developmental Biology and Centro de Investigación Biomédica en Red de Enfermedades Raras (CIBERER), Instituto de Salud Carlos III, Universidad Pablo de Olavide-CSIC-JA, Sevilla, Spain.; 7 Department of Microbiology and Molecular Genetics and; 8 Center for Microvascular Research, University of Pittsburgh, Pittsburgh, Pennsylvania, USA.

**Keywords:** Cardiology, Heart failure, Nitric oxide, Radicals

## Abstract

Sudden cardiac death (SCD) in patients with heart failure (HF) is allied with an imbalance in reduction and oxidation (redox) signaling in cardiomyocytes; however, the basic pathways and mechanisms governing redox homeostasis in cardiomyocytes are not fully understood. Here, we show that cytochrome b5 reductase 3 (CYB5R3), an enzyme known to regulate redox signaling in erythrocytes and vascular cells, is essential for cardiomyocyte function. Using a conditional cardiomyocyte-specific CYB5R3-knockout mouse, we discovered that deletion of CYB5R3 in male, but not female, adult cardiomyocytes causes cardiac hypertrophy, bradycardia, and SCD. The increase in SCD in CYB5R3-KO mice is associated with calcium mishandling, ventricular fibrillation, and cardiomyocyte hypertrophy. Molecular studies reveal that CYB5R3-KO hearts display decreased adenosine triphosphate (ATP), increased oxidative stress, suppressed coenzyme Q levels, and hemoprotein dysregulation. Finally, from a translational perspective, we reveal that the high-frequency missense genetic variant rs1800457, which translates into a CYB5R3 T117S partial loss-of-function protein, associates with decreased event-free survival (~20%) in Black persons with HF with reduced ejection fraction (HFrEF). Together, these studies reveal a crucial role for CYB5R3 in cardiomyocyte redox biology and identify a genetic biomarker for persons of African ancestry that may potentially increase the risk of death from HFrEF.

## Introduction

The healthy heart requires a highly synchronized series of reduction and oxidation (redox) reactions to support the electrical and mechanical demands of beating cardiomyocytes (1). In the aging population, the incidence of heart failure (HF) and sudden cardiac death (SCD) escalates and can be traced, in part, to disturbed redox equilibrium and its sequelae: impaired mitochondrial function and metabolism (2–4), oxidative stress (5), Ca^2+^ mishandling (6), and ventricular fibrillation (VF) (2, 7). Although substantial evidence points toward redox imbalance as an initiator of HF and SCD, the basic mechanisms that maintain redox equilibrium in healthy cardiomyocytes are not fully understood.

Cytochrome b5 reductase 3 (CYB5R3), also known as methemoglobin reductase, is a flavoprotein known for catalyzing 1-electron transfer reactions using reduced NADH as an electron donor. CYB5R3 controls diverse biological functions that are modulated partly by its subcellular compartmentalization on the outer mitochondrial membrane facing the cytosol, the endoplasmic reticulum, and the plasma membrane (8–10). Functionally, membrane-associated CYB5R3 exerts antioxidant properties by modulating plasma membrane coenzyme Q (CoQ) reduction (11–13), heme iron reduction (14–16), elongation and desaturation of fatty acids (17), cholesterol biosynthesis (18), and drug metabolism (19, 20), while the soluble form of CYB5R3 reduces RBC methemoglobin (21, 22). Humans with insufficient CYB5R3 activity suffer from recessive hereditary methemoglobinemia, an incurable disease with a clinical presentation of severe neurological complications and early childhood death (23, 24). While the importance of human CYB5R3 function to development is evident, the significance of CYB5R3 in the cardiovascular system and its potential in human cardiovascular disease has not been fully elucidated.

In the vascular wall, CYB5R3 regulates the redox state of α globin in small artery and arteriolar endothelial cells, controlling nitric oxide (NO) diffusion to vascular smooth muscle cells (VSMCs) (14, 15, 25, 26). More recently, we demonstrated that CYB5R3 governs the soluble guanylyl cyclase (sGC) heme redox state to control intracellular cyclic GMP (cGMP) levels in VSMCs (27, 28). While new functional pathways for CYB5R3 are under investigation, no reports have defined the significance of CYB5R3 in cardiomyocytes or the translational impact of modulating CYB5R3 activity in humans with heart disease. Based on the available evidence, we hypothesized that CYB5R3 expression and activity contribute to redox homeostasis in healthy cardiomyocytes and that decreased CYB5R3 function may lead to worse outcomes in patients with HF.

## Results

### Cardiac hypertrophy increases CYB5R3 expression.

To determine whether cardiac hypertrophy and HF cause changes in CYB5R3 expression, we subjected C57BL6/J male mice to transverse aortic constriction (TAC). TAC caused a significant increase in left ventricular (LV) mass, LV anterior wall thickness, and heart weight–to–body weight ratio at 7, 14, and 27 days after TAC, despite no changes in peak aortic valve velocity changes (Supplemental Figure 1, A–C and F; supplemental material available online with this article; https://doi.org/10.1172/JCI147120DS1). Measurements of systolic function, including ejection fraction (EF) and fractional shortening, showed a significant decline at 14 and 27 days after TAC compared with those of control mice (Supplemental Figure 1, D and E). Next, we measured CYB5R3 protein expression at each time point. We found that CYB5R3 expression significantly increased at 7 days following the initiation phase of hypertrophy (Supplemental Figure 1G).

### Restricted deletion of CYB5R3 in cardiomyocytes causes cardiac hypertrophy and SCD in male, but not female, mice.

**Figure 1:**
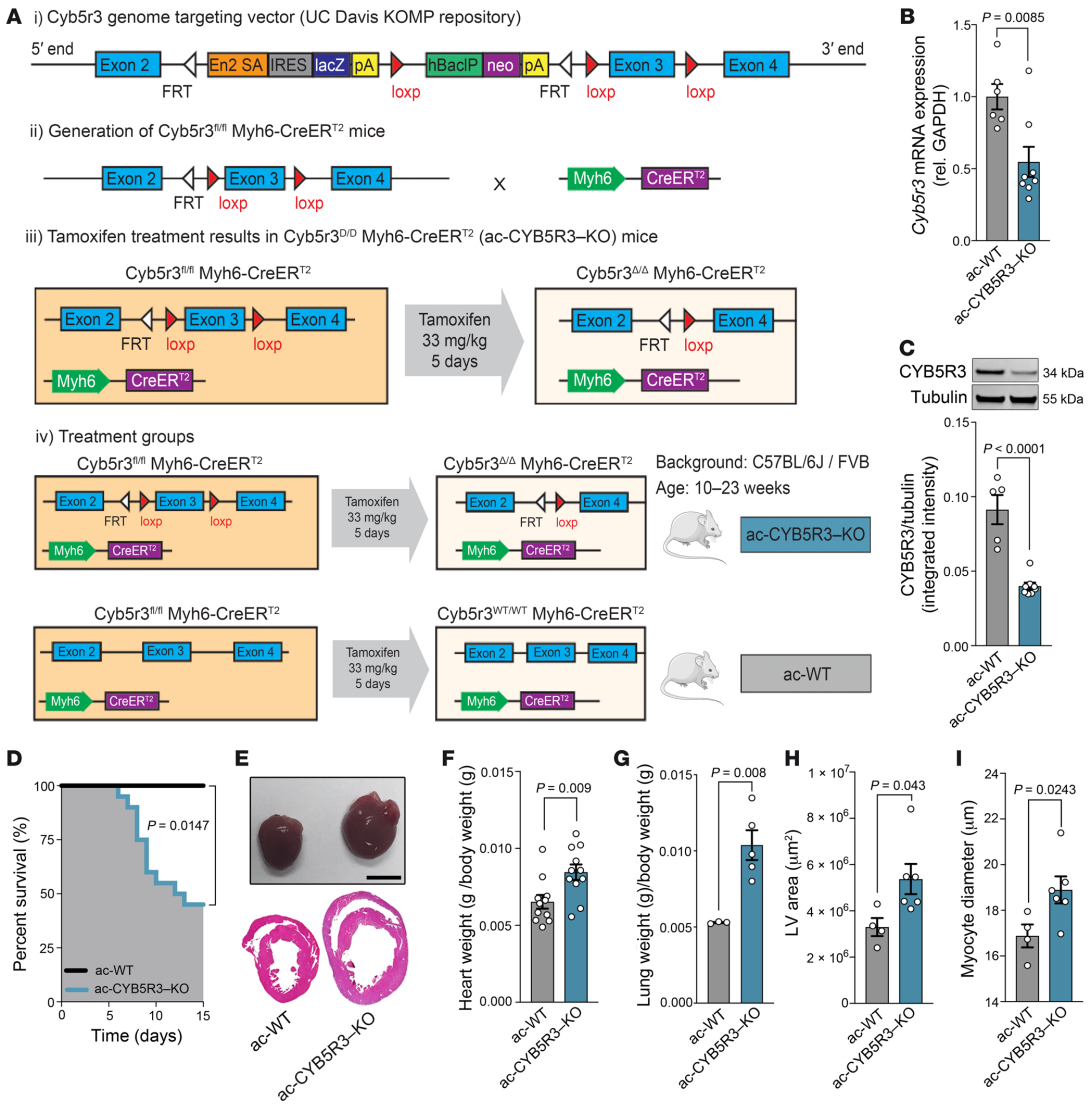
Loss of cardiomyocyte CYB5R3 causes hypertrophy and SCD. (**A**) Detailed schematic showing generation of CYB5R3-KO mice. (**B**) RT-qPCR and (**C**) Western blot analysis of total heart tissue from ac-WT and ac-CYB5R3–KO mice 5 days after tamoxifen injection (*n* = 5–7). (**D**) Survival curve comparing ac-WT (*n* = 8) and ac-CYB5R3–KO mice (*n* = 20). Day 0 represents the first day after tamoxifen injection. (**E**) Gross images (top) and H&E-stained hearts from ac-WT and ac-CYB5R3–KO mice 5 days after tamoxifen injection. Scale bar: 2 mm. (**F**) Heart weight–to–body weight ratio (*n* = 11), (**G**) wet lung weight–to–body weight (*n* = 3–5), (**H**) LV area (*n* = 4–6), and (**I**) myocyte diameter (*n* = 4–6) in ac-WT versus ac-CYB5R3–KO mice. Data are represented as SEM. *P* values were calculated by Student’s *t* test.

To define the functional relevance of CYB5R3 in cardiomyocytes, we generated a tamoxifen-inducible, cardiomyocyte-specific CYB5R3-KO mouse. *Cyb5r3*-floxed mice, as previously described (27), were crossed with tamoxifen-inducible *Myh6-*Cre mice to generate a conditional adult cardiomyocyte CYB5R3 KO (ac-CYB5R3-KO) mouse and an adult cardiomyocyte WT (ac-WT) mouse (Figure 1A). Male ac-CYB5R3-KO and ac-WT mice aged 10 to 13 weeks received tamoxifen injections (33 mg/kg/d) for 5 consecutive days (Figure 1A). We assessed CYB5R3 mRNA and protein from whole heart tissue and found ac-CYB5R3–KO animals had 50% less *Cyb5r3* mRNA expression (Figure 1B) and 70% less CYB5R3 protein (Figure 1C) relative to ac-WT mice at 5 days after tamoxifen treatment. A survival study found 52% of ac-CYB5R3–KO mice died within 15 days after tamoxifen treatment compared with no deaths observed in ac-WT control mice (Figure 1D). Moreover, ac-CYB5R3–KO mice showed biventricular dilation (Figure 1E) and significantly increased heart weight–to–body weight ratios (Figure 1F), lung weight–to–body weight ratios (Figure 1G), LV area (Figure 1H), and myocyte diameter (Figure 1I) relative to their ac-WT counterparts. We found no difference in body weight changes at 3 or 5 days after tamoxifen injection (Supplemental Figure 2, A and B). Additionally, trichrome staining of ac-CYB5R3–KO cardiac tissue at 5 days after tamoxifen injection did not show differences in cardiac fibrosis relative to controls (Supplemental Figure 2, C and D). Complete blood count analysis showed significantly elevated WBC, lymphocyte, and monocyte counts in ac-CYB5R3–KO compared with ac-WT mice (Supplemental Figure 2, E, G, and H). No significant changes to other hematological parameters were detected between groups (Supplemental Figure 2, F and I–N). Notably, loss of CYB5R3 in cardiomyocytes of female mice (Supplemental Figure 3A) did not significantly affect survival (Supplemental Figure 3B), heart weight–to–body weight ratio (Supplemental Figure 3C), or wet lung weight–to–body weight ratio (Supplemental Figure 3D) when compared with that in ac-WTs. All subsequent mouse studies were conducted in male mice.

### Loss of CYB5R3 depresses hemodynamic function and triggers VF.

**Figure 2:**
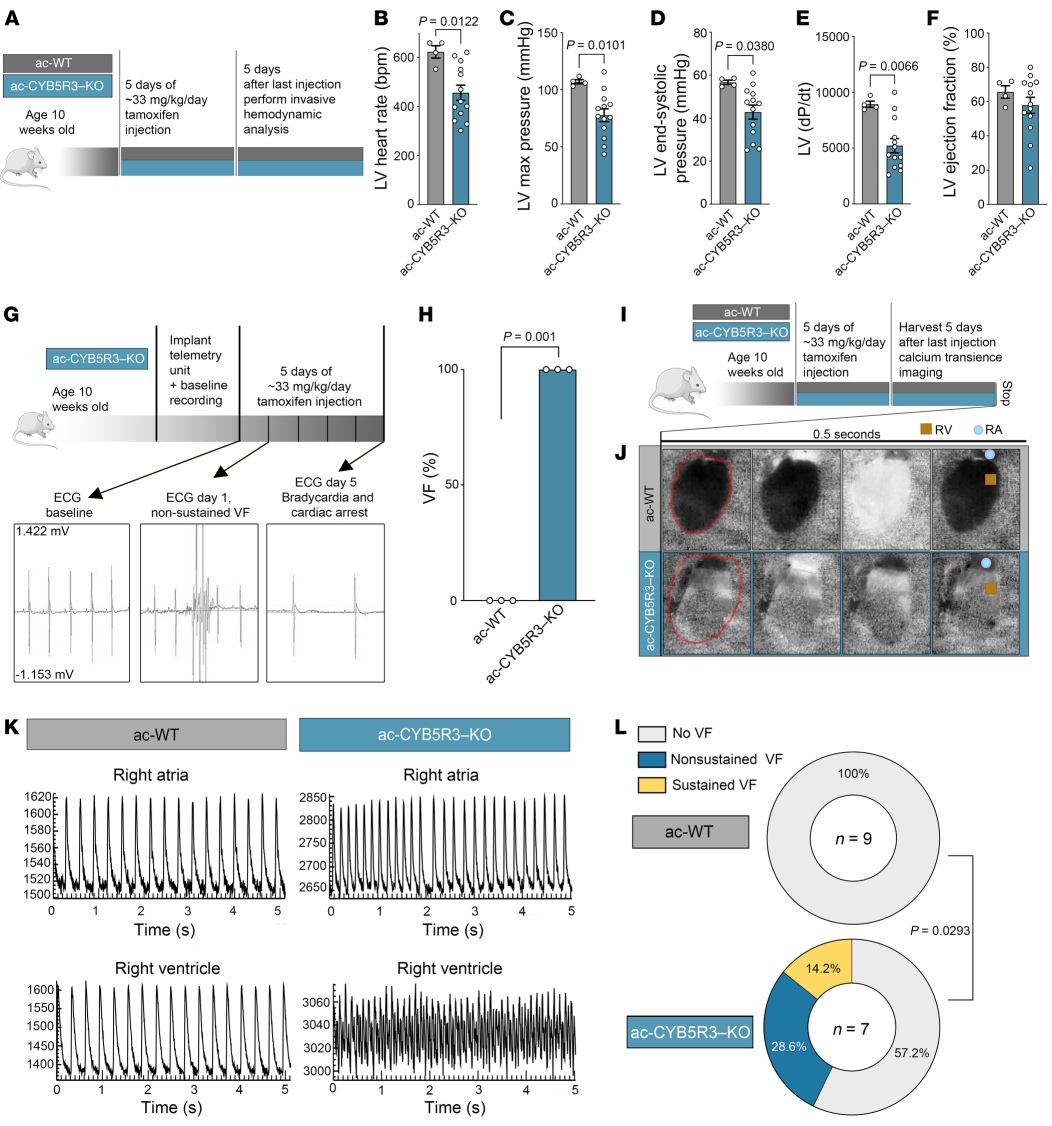
Cardiomyocyte CYB5R3 deficiency leads to VF. (**A**) Experimental design for hemodynamics and measurements of (**B**) LV heart rate, (**C**) LV max pressure, (**D**) LV end-systolic pressure, (**E**) LV *dP/dt*, and (**F**) LV EF (*n* = 4–13). (**G**) Experimental design and ECG measurements and heart rate. (**H**) Quantification of the number of mice with observed VF (*n* = 3). (**I**) ac-WT and ac-CYB5R3–KO mice were injected with 33 mg/kg of tamoxifen for 5 consecutive days. Hearts were isolated and subjected to calcium transient imaging. (**J**) CaTs imaging of ac-WT and ac-CYB5R3–KO mice. Red tracing indicates outline of the heart, and serial images track Ca^2+^ transients over 0.5 seconds. Contrast in the images indicates cytosolic-free Ca^2+^ measured with the calcium indicator dye Rhod2/AM. Regions of interest (blue dots) were drawn on the RA and brown squares on RV to quantify calcium transients. Original magnification, ×2.5. (**K**) Representative tracings of calcium transients of RA and RV of ac-WT and ac-CYB5R3–KO hearts and quantification shown in **L** (*n* = 7–9). Data are represented as SEM. *P* values were calculated by Student’s *t* test (**B**–**F**) and χ^2^ test (**H** and **I**).

To assess cardiac function, we first performed invasive hemodynamic analyses under anesthesia on animals 5 days after tamoxifen injection (Figure 2A). Compared with ac-WT mice, ac-CYB5R3–KO mice showed decreased heart rate, LV maximum pressure, end-systolic pressure, *dP/dt*, and a downward trend for EF (Figure 2, B–F). Next, we conducted radio telemetry studies in conscious animals to measure electrical activity of the heart (ECG) and heart rate. At 24 hours after tamoxifen injections, all ac-CYB5R3–KO mice developed nonsustained VF (Figure 2, G and H). At 5 days after tamoxifen injection, we observed severe bradycardia, leading to cardiac arrest in ac-CYB5R3–KO mice in contrast with ac-WTs (Figure 2G). Next, we measured cardiac cytosolic calcium transients in isolated beating hearts from ac-WT and ac-CYB5R3–KO animals at 5 days after tamoxifen injection using the Langendorff system apparatus preparation (Figure 2I). Hearts were loaded with the calcium indicator dye Rhod-2AM to optically map cardiac calcium transients. Still-frame shots capturing Rhod2 fluorescence bursts (calcium transients) during sinus rhythm were obtained using high-speed imaging (Figure 2J). Distinct atrial-to-ventricular (AV) Ca^2+^ transients (CaTs) were observed in ac-WT animals, shown by large CaTs in right atria (RA), followed by right ventricular (RV) CaTs after AV delay (Figure 2J). Conversely, baseline optical mapping showed that ac-CYB5R3–KO mice developed AV block with out-of-phase AV depolarization and dysregulated ventricular CaTs (Figure 2, J and K). Quantification showed that 42.8% of the ac-CYB5R3–KO hearts exhibited VF, 28.6% with nonsustained VF and 14.2% with sustained VF, while the ac-WT hearts did not show VF (Figure 2L).

### Loss of cardiomyocyte CYB5R3 causes cardiac structural remodeling and bioenergetic depletion.

**Figure 3:**
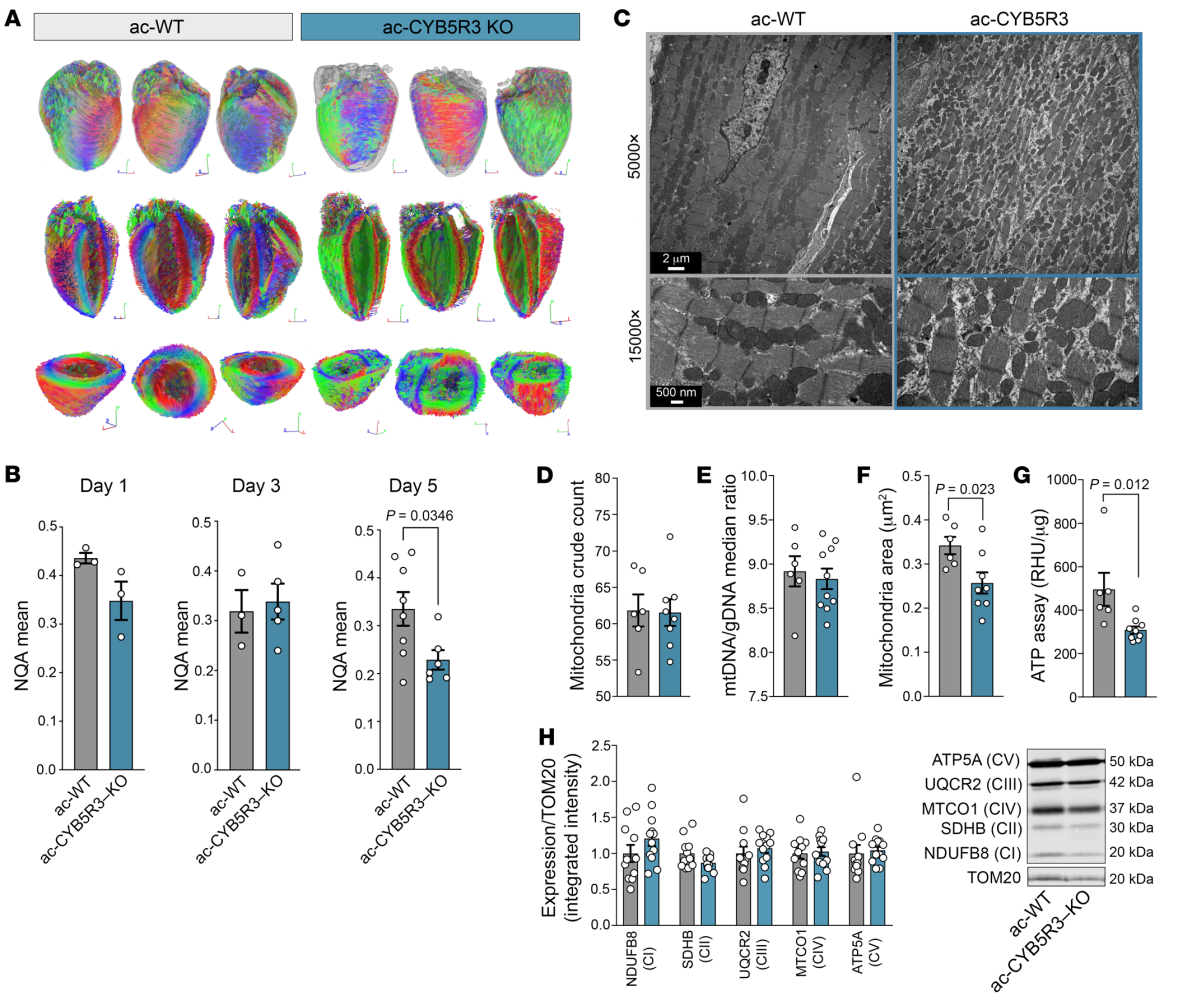
ac-CYB5R3–KO leads to structural remodeling, decreased mitochondrial size, and suppressed ATP levels. (**A**) The representative fiber tractography of ac-WT (left) and an ac-CYB5R3–KO (right) hearts on day 5 after tamoxifen injection. The orientations of the myocardial fibers in relation to the laboratory coordinates are represented by different colors: red representing left-to-right fiber orientation, blue representing front-to-back fiber orientation, and green representing top-to-bottom orientation. Top row: surface viewing of the volume rendition of the myofibers. Gray outlines the heart volumes. Middle row: long-axis 4-chamber cutaway views of the myocardial fibers. Bottom row: short-axis cutaway views of the myocardial fibers. The viewing angle corresponds to the S-L-P axes located at the bottom right of each image. (**B**) NQA measuring the myofiber coherency on posttamoxifen day 1, day 3, and day 5 comparing ac-WT and ac-CYB5R3–KO hearts (*n* = 3–8). (**C**) Representative TEM images of ac-WT (gray, left) and ac-CYB5R3 (blue, right) cardiomyocytes (*n* = 6 and *n* = 8, respectively). Original magnification, ×5000 (top); ×15,000 (bottom). (**D**) Cardiomyocyte mitochondrial count from ×1500 TEM images from ac-WT and ac-CYB5R3–KO mice. (**E**) mtDNA/gDNA ratio from heart tissue of ac-WT and ac-CYB5R3–KO mice (*n* = 6 and *n* = 10, respectively). (**F**) Mitochondrial area from ×15,000 TEM images from ac-WT and ac-CYB5R3–KO hearts. (**G**) Total ATP levels in whole heart lysate from ac-WT and ac-CYB5R3–KO hearts (*n* = 6 and *n* = 9, respectively). (**H**) Western blot of each mitochondrial subunit relative to TOM20 protein expression (control, *n* = 11; CYB5R3-KO, *n* = 12). Data are represented as SEM. *P* values were calculated by Student’s *t* test.

Myofiber architecture was evaluated using diffusion tractography derived from diffusion tensor imaging (DTI) (29). Figure 3A shows diffusion tractography of myocardial fibers in ac-WT and ac-CYB5R3–KO hearts at 5 days after tamoxifen injection with volume rendering, long-axis cutaway views, and short-axis cutaway views. The 3D rendition of diffusion tractography of the ac-WT control heart showed ordered myocardial fiber organization throughout the heart layers. In contrast, the ac-CYB5R3–KO heart (Figure 3A) displayed disarrayed myocardial fibers. The degree of myofiber disarray progressed over time, as shown by normalized quantitative anisotropy (NQA) on days 1, 3, and 5 (Figure 3B) after tamoxifen injection. On day 5, the ac-CYB5R3–KO hearts (Figure 3A) showed significantly reduced NQA compared with their ac-WT counterparts (Figure 3A). To corroborate these observations, we conducted ultrastructure studies using transmission electron microscopy (TEM). Consistent with DTI, we observed myocardial disorganization in ac-CYB5R3–KO hearts relative to ac-WT (Figure 3C). These structural changes were independent of apoptosis measured by TUNEL staining (Supplemental Figure 4, A and B). Mitochondria number, as determined by both mitochondrial crude count (Figure 3D) from TEM images and mitochondrial DNA to genomic DNA ratio (mtDNA/gDNA) (Figure 3E), were similar between ac-CYB5R3–KO and ac-WT hearts. On the other hand, mitochondrial area (Figure 3F) was 30% lower in ac-CYB5R3–KO hearts and was accompanied by significantly lower total adenosine triphosphate (ATP) (Figure 3G) relative to ac-WT hearts. Additionally, we found no difference in autophagy or mitophagy measured via LC3II/LC3I ratio or changes in multimembrane structures at the ultrastructure level between groups (Supplemental Figure 5, A–C). Protein measurements of the outer mitochondrial membrane transporter TOM20 and mitochondrial respiratory complex subunits were similar between groups (Figure 3H).

### Bioenergetic, metabolic, and muscle contractility pathways are altered in ac-CYB5R3–KO mice.

**Figure 4:**
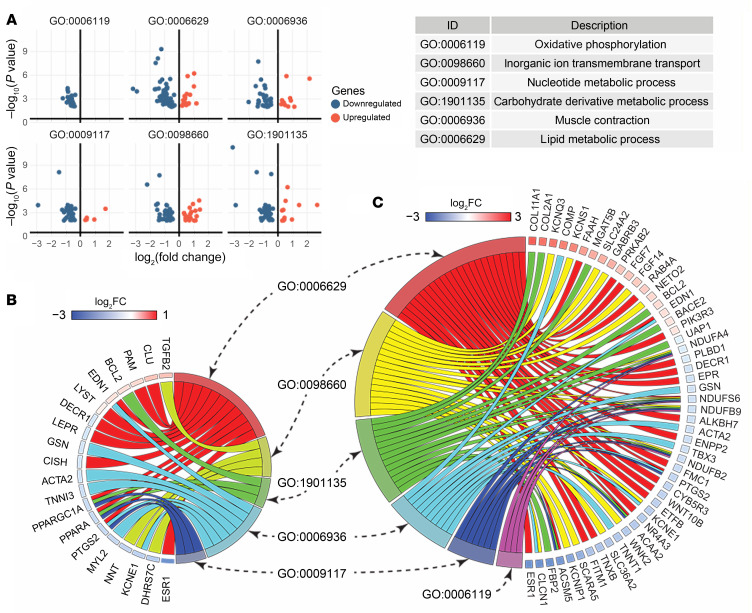
RNA-Seq analysis shows ac-CYB5R3–KO induces transcriptome changes enriched in metabolism, oxidative phosphorylation, and muscle contraction pathways. (**A**) GO pathway analysis showed differential expression of genes (*P* < 0.01) in pathways (adjusted *P* < 0.05) involved in metabolism and muscle contraction. (**B**) Genes overrepresented in HF (DisGeNET C0018801, adjusted *P* = 7.820 × 10^–7^) were mapped to the selected metabolism and muscle contraction pathways. (**C**) Top differential expression genes (adjusted *P* < 0.02) are mapped to the same GO pathways.

To better understand the signaling pathways preceding the pathological changes in ac-CYB5R3–KO mouse hearts, we performed RNA-Seq experiments on whole hearts isolated from ac-WT and ac-CYB5R3–KO mice 5 days after tamoxifen injection. Pathway analysis using the Gene Ontology (GO) database for biological processes identified differentially expressed genes (*P* < 0.01) between ac-CYB5R3–KO and ac-WT mouse hearts as being significantly enriched (adjusted *P* < 0.05) in pathways regulating oxidative phosphorylation, ion transport, muscle contraction, and metabolism involving nucleotides, carbohydrates, and lipids (Figure 4A). Within these pathways, we found a greater portion of genes to be downregulated rather than upregulated in the ac-CYB5R3–KO hearts compared with the ac-WT hearts (Figure 4A). Additionally, differentially expressed genes are significantly associated with HF (DisGeNET C0018801, adjusted *P* = 7.820 × 10^–7^). Notably, many genes are shared by the abovementioned pathways, suggesting that aberrant signaling in mitochondrial respiration, cellular metabolism, and muscular contractility is responsible for the pathological changes in CYB5R3-deficient hearts (Figure 4B). Furthermore, we found over one-third of most differentially expressed genes (adjusted *P* < 0.02) were also associated with these pathways (Figure 4C). Ranked by fold change, the most affected genes were iron-regulatory genes, such as α synuclein (*Snca*) and 5′-aminolevulinate synthase (*Alas2*), which is consistent with CYB5R3’s role in regulating iron redox states (Supplemental Table 3).

### Oxidative stress is increased in hearts following cardiomyocyte CYB5R3 deletion.

**Figure 5:**
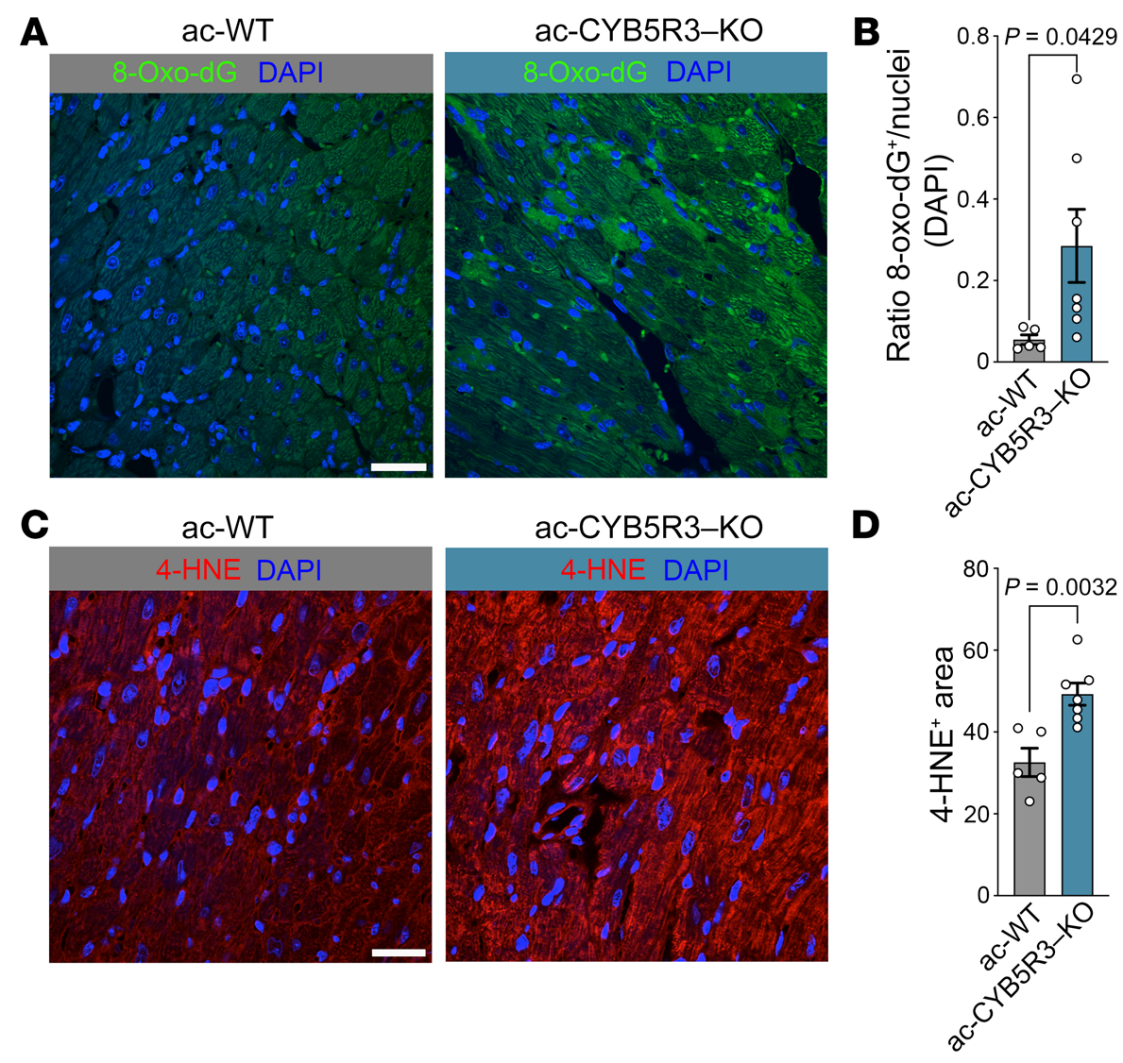
Cardiac-specific KO of CYB5R3 increases 8-oxoG and 4-HNE staining. Heart sections were stained for 8-oxoG and 4-HNE measurements of oxidative stress. (**A**) Representative image of control (ac-WT, gray, *n* = 5) and KO (ac-CYB5R3–KO, blue, *n* = 7) heart sections stained for 8-oxoG (green) and DAPI (blue). (**B**) Quantification of total 8-oxoG puncti relative to DAPI puncti. (**C**) Representative ac-WT (gray, *n* = 5) and ac-CYB5R3–KO hearts (blue, *n* = 7) stained for DAPI (blue) and 4-HNE (red). (**D**) Integrated intensity of 4-HNE staining. Data are represented as SEM. *P* values were calculated by Student’s *t* test. Scale bars: 50 μm.

Based on prior work demonstrating that CYB5R3 is a common effector of the nutritional and oxidative stress responses (30), we measured oxidative stress levels in ac-CYB5R3–KO and ac-WT control hearts by 8-oxoguanine (8-oxoG) (Figure 5, A and B) and 4-hydroxynonenal (4-HNE) staining (Figure 5, C and D). Hearts from ac-CYB5R3–KO mice exhibited more cells positive for 8-oxoG and 4-HNE staining than ac-WT control hearts, indicating more oxidized guanine bases in DNA and greater lipid peroxidation, respectively.

### Ubiquinol, the reduced form of CoQ, is decreased in hearts with CYB5R3 KO.

**Figure 6:**
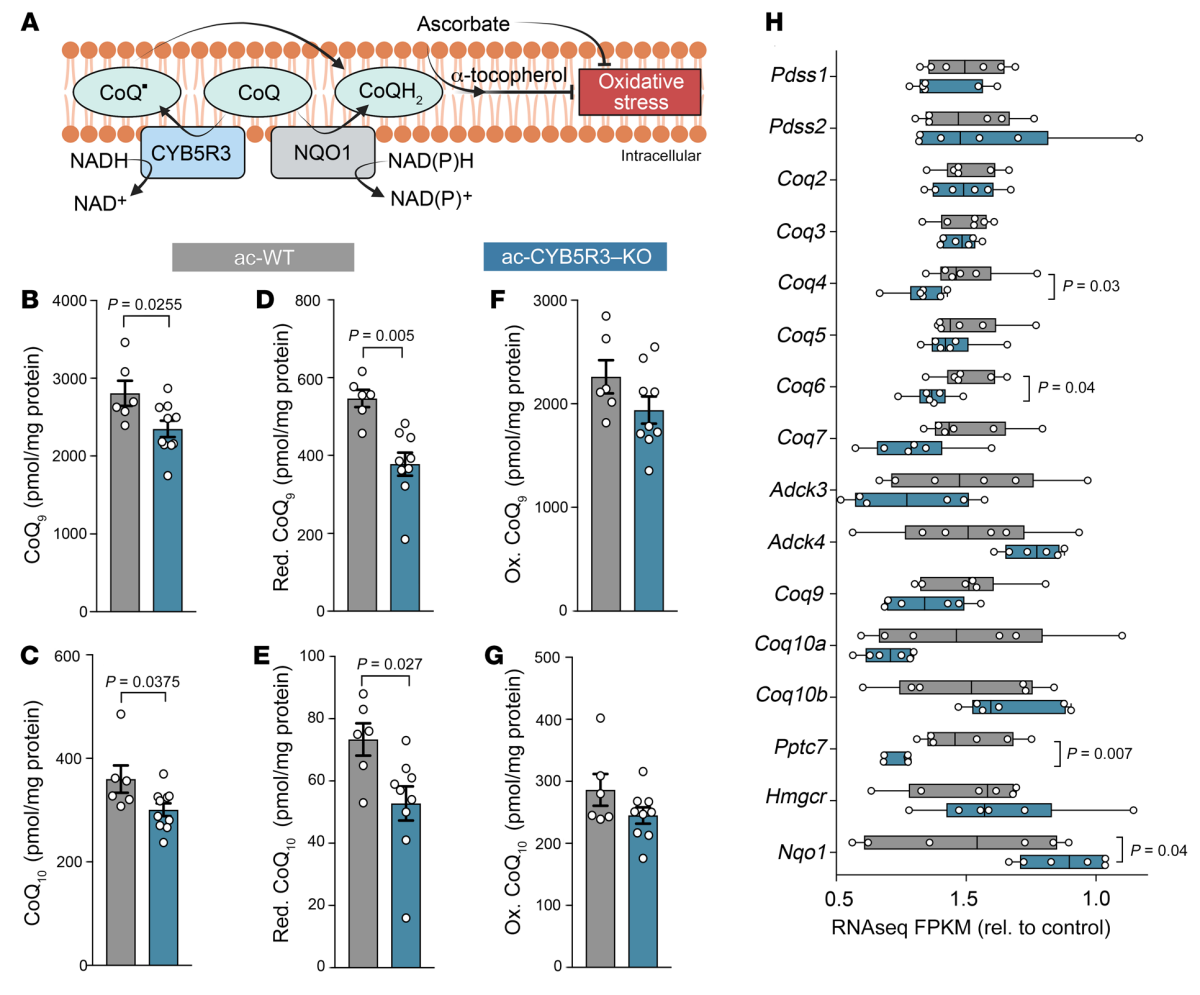
Loss of cardiac CYB5R3 decreases total and reduced levels of ubiquinone. Whole heart tissue from control (ac-WT, gray, *n* = 6) and KO (ac-CYB5R3–KO, blue, *n* = 9) mice was used to assess the role of cardiac CYB5R3 in CoQ reduction. (**A**) Schematic of CYB5R3’s known role in the plasma membrane redox system as a CoQ reductase. Concentration of (**B**) total CoQ_9_, (**C**) total CoQ_10_, (**D**) reduced CoQ_9_, (**E**) reduced CoQ_10_, (**F**) oxidized CoQ_9_, and (**G**) oxidized CoQ_10_. (**H**) Data extracted from RNA-Seq showing relative expression of CoQ synthesis genes and Nqo1, a known redox regulator of CoQ. Data are represented as SEM. *P* values were calculated by Student’s *t* test.

CYB5R3 in the plasma membrane is known to work in conjunction with NAD(P)H quinone dehydrogenase 1 (NQO1) for 1- and 2-electron reduction of the oxidized form of CoQ (ubiquinone), protecting membranes from overt oxidative damage (Figure 6A) (11–13). Therefore, we asked whether loss of cardiomyocyte CYB5R3 altered total CoQ levels and their redox state in hearts. Using high-performance liquid chromatography with electrochemical detection (HPLC-ECD), we found 30% less reduced CoQ_9_ and CoQ_10_ in ac-CYB5R3–KO hearts relative to ac-WT controls (Figure 6, B and C). Total CoQ loss was linked to a greater loss of reduced CoQ (Figure 6, D and E) than oxidized CoQ (Figure 6, F and G). Next, we measured gene changes in the CoQ synthesis pathway and found significant decreases in *CoQ4*, *CoQ6*, and *Pptc7* mRNA expression and a significant increase in the CoQ redox cycling gene *Nqo1* (Figure 6H).

### Lipid composition of cardiomyocyte cell membranes is altered with CYB5R3 KO.

CYB5R3 is known to regulate lipid metabolism in the liver (31), but its effects on myocardial lipid metabolism are unknown. Lipids are critical to the physical properties of cell membranes and many of their functions, including recruitment of intracellular proteins to membrane surfaces, enzymatic activity of transmembrane proteins, signaling of lipid second messengers, and fusion of membranes (32). Additionally, they serve as lipid reservoirs of arachidonate to support the synthesis of lipid signaling mediators via cyclooxygenase and lipoxygenase activity (e.g., eicosanoids). Therefore, HPLC–tandem mass spectrometry (HPLC-MS/MS) was used to determine the impact of decreased CYB5R3 on the cell membrane lipid profile of total heart tissues. Most phosphatidylcholine (PC) species did not change upon loss of CYB5R3; ac-CYB5R3–KO and ac-WT cardiomyocytes exhibited similar distribution and content of PC species, except for PC containing linoleic and arachidonic acids in their *S_N_2* position (Supplemental Figure 6, A–D). Significant decreases ranging from 30% to 42% were observed in 1-stearoyl- and 1-palmitoyl-2-linoleoyl–PC and 1-stearoyl- and 1-palmitoyl-2-arachidonoyl–PC in the ac-CYB5R3–KO cardiomyocytes relative to WT controls (Supplemental Figure 6, E–H). A 50% decrease in nuclear stearoyl-arachidonate PC and phosphatidylethanolamine has been previously reported in rat cardiomyocytes following ischemia-reperfusion injury (33). However, lysophosphatidylcholine (LysoPC) levels were similar between the ac-CYB5R3–KO and ac-WT groups (Supplemental Figure 6, I–O).

### cGMP and PKG signaling in hearts is diminished with CYB5R3 KO.

Our previous work demonstrated that loss of CYB5R3 from VSMCs affects the heme redox state of sGC β and downstream PKG signaling (27, 28). We found ac-CYB5R3–KO heart tissue had significantly less cGMP content (Supplemental Figure 7A) and diminished PKG activity assessed by PKG-dependent phosphorylation of serine 239 of the vasodilator-stimulated protein (VASP) (Supplemental Figure 7B), but similar sGC protein expression levels relative to those of ac-WT controls (Supplemental Figure 7C). Finally, quantitative reverse-transcription PCR (qRT-PCR) showed a 50% decrease in expression of *Ppargc1a*, a master regulator of mitochondria biogenesis, in ac-CYB5R3–KO hearts relative to ac-WT controls (Supplemental Figure 7D), a result consistent with decreased gene expression shown in the RNA-Seq analysis in Figure 4B.

### Oxygen supply is diminished in cardiomyocytes with CYB5R3 KO.

**Figure 7:**
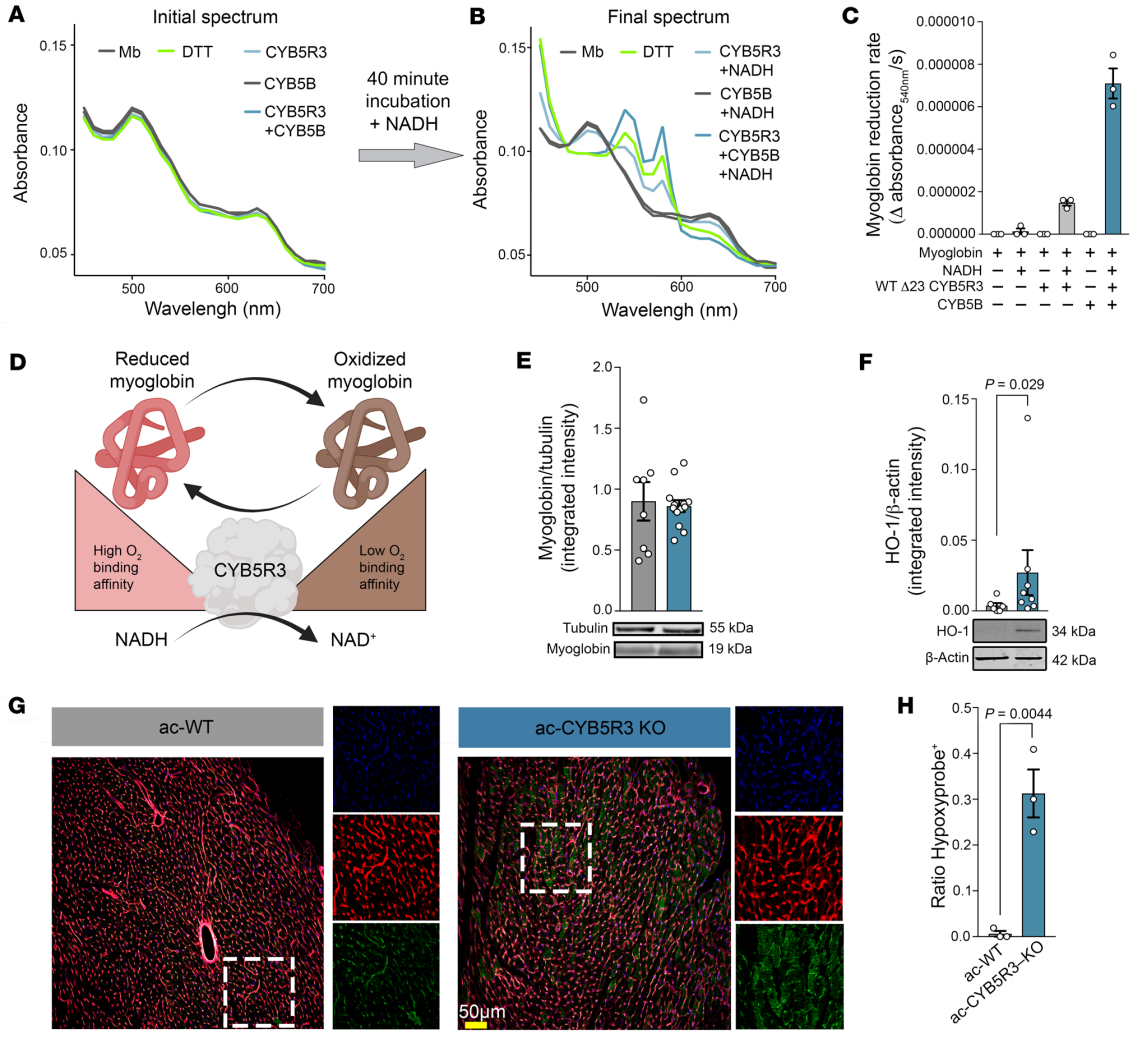
Cardiomyocyte CYB5R3 is critical for myoglobin reduction and maintenance of intracellular oxygen tension. (**A**) In vitro purified colorimetric assay showing absorbance shift of oxidized myoglobin to reduced myoglobin (**B**) after the addition of electron donor NADH. (**C**) Rate of absorbance changes at 540 nm, with observation of increasing amounts of reduced myoglobin over time. (**D**) Schematic of hypothesized redox regulation of myoglobin by CYB5R3 and the relevant impact on oxygen binding. (**E**) Western blot of heart lysates measuring myoglobin and β-actin as a loading control with quantification of relative integrated intensities (ac-WT, *n* = 8; ac-CYB5R3–KO, *n* = 13). (**F**) HO-1 and β-actin loading control Western blot with quantification of relative integrated intensities. (**G**) Hypoxyprobe staining (green) of heart sections from control (ac-WT *n* = 3) and Cyb5R3-KO (ac-CYB5R3–KO, *n* = 3) heart sections, counterstained with wheat germ agglutinin (red) and DAPI (blue). Original magnification, ×3 (right panels). (**H**) Quantification of Hypoxyprobe-positive cells relative to total cells. Data are represented as SEM. *P* values were calculated by Student’s *t* test. Scale bar: 50 m.

To determine whether CYB5R3 reduces myoglobin, an abundant heme protein that facilitates oxygen delivery to mitochondria in cardiomyocytes, we synthesized recombinant CYB5R3 and cytochrome B5B (CYB5B), the latter being a small heme protein that facilitates electron transfer from CYB5R3. Using ultraviolet-visible (UV-VIS) spectrophotometry, we found that NADH catalyzed reduction of oxidized myoglobin (absorbance change at 540 nm) by CYB5R3 and CYB5B (Figure 7, A–C), which is consistent with previous work (16). Dithiothreitol (DTT) served as a positive control. These in vitro data support the idea that CYB5R3 may serve as a myoglobin reductase in vivo. Since reduced myoglobin heme iron facilitates oxygen storage and diffusion in cardiomyocytes only in its reduced form (Figure 7D) (34–36), we assessed intracardiac oxygenation status in ac-CYB5R3–KO and ac-WT mice using Hypoxyprobe, which adducts to cysteine residues at tissue sites with less than 10 mmHg pO_2_. Following Hypoxyprobe injection, hearts from ac-CYB5R3–KO mice were isolated to assess levels of myoglobin, the heme-degrading enzyme heme-oxygenase-1 (HO-1), and Hypoxyprobe. All hearts exhibited similar levels of myoglobin (Figure 7E), but hearts from ac-CYB5R3–KO mice showed HO-1 upregulation by 4-fold (Figure 7F) and significantly more Hypoxyprobe staining than ac-WT hearts (Figure 7, G and H). Given these changes occurred independently of myoglobin protein expression (Figure 7E), these data link heme oxidation and release, possibly from myoglobin, with loss of CYB5R3.

### CYB5R3 T117S is enriched in end-stage HF ventricular tissue from African American patients and is associated with decreased event-free survival.

**Figure 8:**
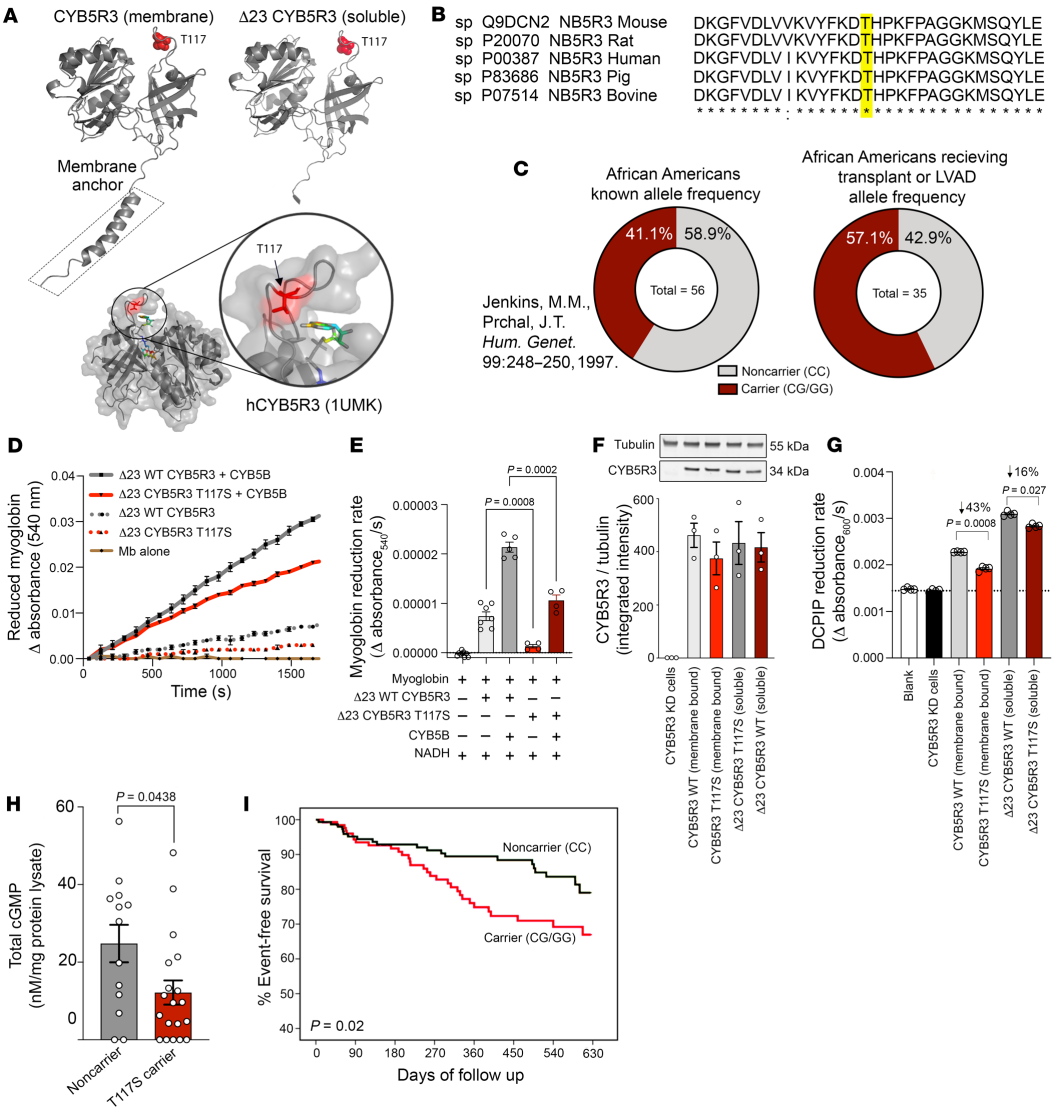
CYB5R3 T117S is a partial loss-of-function variant that associates with accelerated death in African American HF patients. (**A**) Structure of membrane and soluble CYB5R3. Red colored area shows T117 residue (dark red) of the reductase. Below, the green-blue-yellow figure shows the FAD prosthetic group of the reductase. (**B**) Amino acid sequence aligned across mammals showing conserved threonine (highlighted in yellow) in the 117 position of membrane CYB5R3. (**C**) Comparison of known allele frequency of T117S in African Americans (left pie chart) compared with allele frequency of T117S in African Americans with HF that received transplant or LV assist devices (LVAD). Gray represents the proportion of noncarriers relative to red region representing pooled heterozygous and homozygous CYB5R3 T117S carriers. (**D**) Reduced myoglobin over time in vitro comparing 23 T117S (soluble) with WT 23 CYB5R3 (soluble) with and without CYB5B. (**E**) Change in myoglobin reduction rates detected at 540 nm/s (*n* = 4–9). (**F**) Western blot of reexpressed CYB5R3 WT, CYB5R3 T117S, 23 CYB5R3 WT, and 23 CYB5R3 T117S in HEK293 FT CYB5R3-KO≈cells (*n* = 3). (**G**) CYB5R3 activity over time measured by 2,6-dichlorophenolindophenol (DCPIP) reduction, comparing CYB5R3 WT, CYB5R3 T117S, 23 CYB5R3 WT, and 23 CYB5R3 T117S (*n* = 3). (**H**) Total cGMP in heart lysates from African Americans receiving ventricular assist devices, comparing noncarriers (gray) and T117S carriers (red) (*n* = 13–19). (**I**) Event-free survival curve comparing African American CYB5R3 T117S CC versus CG/GG from the GRACE and GRAHF trials. Data are represented as SEM. *P* values were calculated by Student’s *t* test (**G** and **H**), 1-way ANOVA (**E**), or a log-rank test (**I**).

Over 40 genetic polymorphisms in CYB5R3 have been identified (23). The missense variant rs1800457 translates into the CYB5R3 T117S mutation in the membrane-bound form expressed in somatic cells (Figure 8A) wherein threonine is substituted with serine. This is a high-frequency genetic variant in individuals with African ancestry (23% minor allele frequency that occurs with less than 1% frequency in other ethnicities; refs. 37–40). Based on x-ray crystallography (Protein Data Bank [PDB] structure 1UMK), T117S was positioned in a cytosolic-facing loop near the flavin (FAD) group,which is essential for electron transfer from NADH (Figure 8A). This threonine is conserved across many species, including human, mouse, rat, and pig, suggesting that this site is evolutionarily important (Figure 8B). To determine whether this variant is associated with HF with reduced EF (HFrEF), we genotyped 35 heart tissue samples collected from African American patients at the time of cardiac transplant or LV assist device (LVAD) implantation at the University of Pittsburgh Medical Center (Pittsburgh, Pennsylvania, USA). Baseline demographics are shown in Supplemental Figure 8. We found that the allele frequency increased in this cohort from 41.1% to 57.1% (Figure 8C). Using recombinant 23 CYB5R3 T117S (T94S) purified protein, we tested the mutation’s impact on reductase activity. An in vitro activity assay showed that recombinant 23 CYB5R3 T117S reduced oxidized myoglobin at 40% of the rate of WT CYB5R3 (Figure 8, D and E). Coincubation with CYB5B significantly increased the rate of oxidized myoglobin reduction by 23 CYB5R3 T117S, although leaving it 40% slower than WT CYB5R3 with CYB5B. To determine whether the membrane anchor region of CYB5R3 affects catalytic activity in lysates, we transfected CYB5R3-KO HEK293 cells with either membrane-bound CYB5R3 (T117S) or soluble CYB5R3 (T94S or 23 CYB5R3 T117S) (Figure 8F). We found that both membrane-associated and soluble CYB5R3 had markedly less activity compared with WT (Figure 8G). Since CYB5R3 regulates cGMP levels, we measured ventricular cGMP levels in HF patients of African-American ancestry. In these patients, we found 50% less cGMP in the carriers of the T117S variant than the noncarriers (WT CYB5R3 form) (Figure 8H). We next extended these studies with a meta-analysis to determine the association of CYB5R3 T117S with event-free survival in self-identified Black individuals from the Genetic Risk Assessment of Heart Failure (GRAHF) study in African Americans, a genetic substudy of the African American Heart Failure Trial (AHeFT) (ClinicalTrials.gov NCT00047775) (41, 42), and the Genetic Risk Assessment of Cardiac Events (GRACE) study, a single-center genetic outcomes registry from the University of Pittsburgh Medical Center (43, 44). For the combined outcome analysis, subjects receiving fixed dose combination therapy of isosorbide dinitrate and hydralazine (FDC I/H) were excluded. Subjects were followed prospectively until an endpoint of death, cardiac transplantation, or HF hospitalization. Baseline demographics for CYB5R3 WT (CC) versus T117S carriers (both heterozygotes [CG] and homozygotes [GG]) are shown in Supplemental Table 4. We found that T117S carriers had significantly reduced event-free survival (*P* = 0.02) over a 1.5-year period when compared with noncarriers, despite being given the same standard HF therapy (beta blockers, ACE inhibitors, and aldosterone antagonists) (Figure 8I).

## Discussion

**Figure 9:**
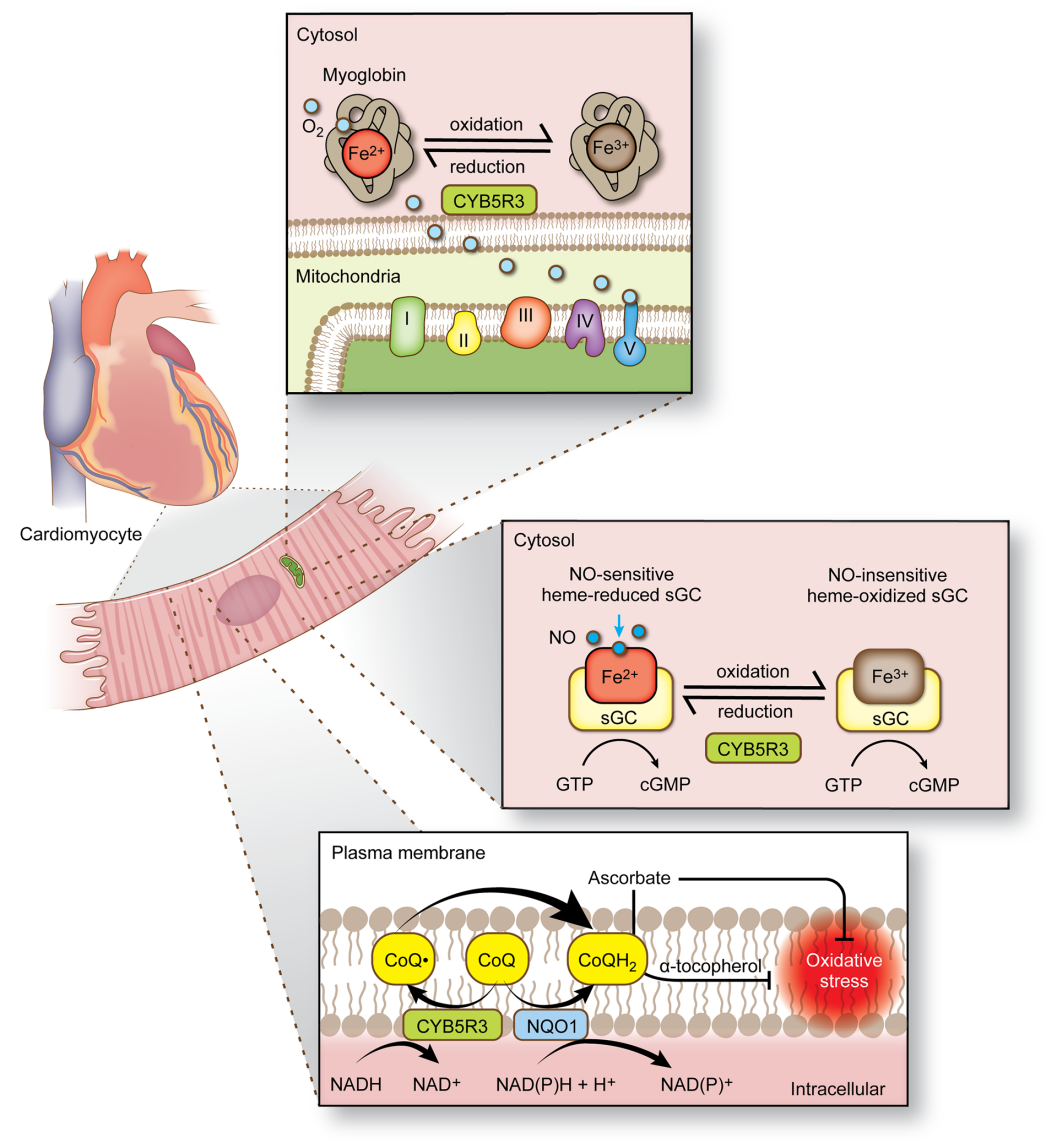
Schematic overview of cardiomyocyte CYB5R3 function. The upper panel shows that CYB5R3 reduces heme-bound myoglobin to facilitate oxygen (light blue circles) diffusion to mitochondria. The middle panel shows that CYB5R3 reduces sGC heme to enable NO (dark blue circles) binding and production of cGMP. The lower panel shows that CYB5R3 and NQO1 coordinately reduce membrane-embedded CoQ to mitigate oxidative stress via α-tocopherol.

Despite better health management in the aging population and emerging treatments for HF, patient prognosis remains poor, with nearly 20% of patients dying within the first year of diagnosis and the remainder dying within the next 7 years (45–47). Estimates indicate that SCD occurs 6- to 9 -fold more often in HF patients compared with the general population (48). While patients with HF present with structural remodeling and cardiac electrical changes that can often lead to lethal cardiac arrhythmias, there is a growing consensus that SCD most likely stems from upstream signaling events that cause electrical variability and ventricular dysfunction (49). Allied to these upstream signaling events is redox imbalance that can lead to impaired mitochondrial function and metabolism (2–4), oxidative stress (5), and Ca^2+^ mishandling (6). This study provides evidence that loss of CYB5R3 expression in adult male mouse cardiomyocytes causes SCD that is associated with Ca^2+^ mishandling, increased oxidative stress, decreased ATP production, and loss of redox regulation of myoglobin and CoQ, indicating that CYB5R3 is essential for maintaining cardiac redox equilibrium (Figure 9). From a translational perspective, we ascertain that the high-frequency missense genetic variant CYB5R3 T117S associates with a decreased event-free survival (~20%) in Black persons suffering from HFrEF. Together, these findings highlight the functional significance of CYB5R3 in cardiomyocyte biology and a genetic variant that may identify Black patients with HFrEF for increased risk of poor outcomes.

CYB5R3 is highly compartmentalized at the subcellular level, where its expression is enriched on membranes of mitochondria, endoplasmic reticulum, and the plasma membrane. Localization of CYB5R3 to membranes is largely regulated by *N*-myristoylation (8). Membrane-bound CYB5R3 contributes to a plethora of functions in somatic cells, including reduction of heme iron (14–16), elongation and desaturation of fatty acids (17), biosynthesis of cholesterol (18), and hepatic metabolism of drugs (19, 20). In fibroblasts, membrane-bound CYB5R3 has been shown to be a common effector of nutritional and oxidative stress responses through the transcription factors FOXO3a and Nrf-2 as well as a modulator of lipid metabolism (30). Aging studies have shown that CYB5R3 expression decreases with aging and CYB5R3 overexpression extends life and health spans (10, 30, 31, 50–52). With regard to the cardiovascular system, growing evidence supports a critical role for CYB5R3 in the vascular wall. We recently showed that CYB5R3 regulates the heme redox state of hemoglobin α in small artery and arteriolar endothelial cells, controlling NO diffusion to VSMCs (14). Additionally, we uncovered that CYB5R3 sensitizes sGC to NO by reducing sGC heme iron, functioning as a control mechanism for intracellular cGMP levels in VSMCs (27, 28, 53). While these reports show that CYB5R3 modulates oxidative stress, mitochondrial function, CoQ redox regulation, and NO/cGMP signaling in a diverse number of cell types, the data provided herein support a central role for CYB5R3 as a “redox hub” in cardiomyocytes, governing independent redox signaling pathways in a unified manner. Hence, the etiology that initiates, propagates, and exacerbates cardiac dysfunction and associated SCD in the ac-CYB5R3–KO mice most likely stems from a multitude of dysregulated redox-signaling functions regulated by CYB5R3 in the cardiomyocyte.

In male ac-CYB5R3–KO mice, we observed a rapid onset of SCD that was associated with abnormal Ca^2+^ handling, cardiomyocyte hypertrophy, structural reorganization, ATP depletion, and VF. These data likely point to VF and bradycardia as causes for SCD in these mice; however, instigation of Ca^2+^ mishandling is likely propagated through a series of interrelated redox signaling events. Based on published data and data shown here, we and others have found that CYB5R3 controls redox balance through its ability to reduce 2 key substrates: (a) CoQ_10_ (CoQ→CoQH_2_), a membrane-bound molecule needed for electron transport and tempering oxidative stress, and (b) heme (Fe^3+^→Fe^2+^), an iron-centered protoporphyrin ring that is an essential cofactor required for binding of oxygen to myoglobin and NO to sGC so that cGMP can be produced.

CoQ is an enriched, lipid-soluble molecule embedded in the inner mitochondrial membrane, where it participates in electron transfer reactions to support oxidative phosphorylation needed for cellular ATP production. Except for the inner mitochondrial membrane, the role of CoQ is largely undefined in organelles (Golgi, endoplasmic reticulum, and outer mitochondrial membranes), where CYB5R3 can localize via posttranslational modifications (8). Outside the cardiovascular system, plasma membrane-bound CYB5R3, in conjunction with NQO1, can reduce CoQ and suppress oxidative stress via α-tocopherol and ascorbate (11–13), a system known as the “plasma membrane redox system” (12, 13). Our data support these previous observations and show that ac-CYB5R3–KO hearts exhibit significantly decreased levels of reduced and total CoQ_9_ and CoQ_10_ (total CoQ_9_ and CoQ_10_: *P* = 0.0255, *P* = 0.0375, respectively; reduced CoQ_9_ and CoQ_10_: *P* = 0.005, *P* = 0.027, respectively) as well as reduced *coq4*, *coq6*, and *Pptc7* gene expression. Moreover, we found reduced mitochondrial size, loss of linoleoyl- and arachidonoyl-PC species, elevated oxidative stress, and ATP depletion. Based on previous work, it is plausible that CYB5R3 plays an active role in reducing CoQ and protecting membranes from oxidative damage. Studies showing reduced mitochondrial area in the ac-CYB5R3–KO mice may suggest increased mitochondrial membrane damage due to oxidative stress and loss of membrane integrity, allowing damaged mitochondria to promote fragmentation of healthy mitochondria and decreased ATP levels. However, we did not observe any changes in LC3II/I expression or increased multimembrane structures indicative of autophagy or mitophagy in cardiomyocytes. While our studies focused on the 5 days after tamoxifen time point, it is possible that earlier time points may show differences. Additionally, impaired mitochondrial biogenesis, which can be controlled via NO signaling, may also contribute to decreased ATP. It has previously been reported that NO signaling can regulate mitochondrial biogenesis via stimulation of its receptor, sGC (54). NO-stimulated sGC generates the secondary messenger cGMP, which activates PKG and upregulates the mitochondrial biogenesis master regulator PGC1α (55). Our findings show that loss of cardiomyocyte CYB5R3 significantly decreased cGMP levels (*P* = 0.0438), which was associated with a significantly decreased phospho-VASP/VASP ratio (a surrogate marker of PKG activity) and suppressed *Pgc1α* transcript levels. While downregulation of mitochondrial complex subunits was not observed at the protein level, RNA-Seq revealed that mRNA levels of 25 mitochondrial complex subunits were downregulated. These results would be consistent with previous reports showing that mitochondrial biogenesis is disrupted in end-stage and ischemic HF (56). Future studies defining the role of CYB5R3 in outer-mitochondrial membrane CoQ reduction relative to its role in other membranes, such as the plasma membrane and endoplasmic reticulum, could provide key insights into the significance of CYB5R3 in outer mitochondrial membrane regulation.

In addition to reducing CoQ in membranes, CYB5R3 reduces oxidized heme, which consists of a protoporphyrin ring with iron in the center for biogas binding and regulation. In RBCs, the soluble form of CYB5R3 reduces methemoglobin (Fe^3+^→Fe^2+^) to enable hemoglobin binding to oxygen (21, 22). In somatic cells, CYB5R3 is critical for reducing sGC heme (Fe^3+^→Fe^2+^), permitting NO binding, sGC activation, and production of cGMP (27, 28). A previous study has demonstrated that, in addition to reducing CoQ and sGC, recombinant CYB5R3 can reduce metmyoglobin, a highly abundant monomeric heme protein in the heart that is critical for oxygen diffusion to mitochondria (16). Cardiomyocyte oxygen tension is known to strongly associate with cardiac performance (57). While previous work disclosed that embryonic deletion of cardiomyocyte-expressed myoglobin is not lethal in mice and does not affect oxygen tension (58), multiple compensatory mechanisms were observed, including increased coronary flow reserve, capillary density, and hematocrit (58). Interestingly, ac-CYB5R3–KO mice subjected to Hypoxyprobe showed reduced cardiomyocyte oxygen tension compared with ac-WTs. These data imply that CYB5R3 is critical for modulating oxygen tension in cardiomyocytes, acting by serving as a reductase of metmyoglobin so that reduced myoglobin can facilitate oxygen delivery to mitochondria. It is important to note that metmyoglobin formation is likely driven by oxidative stress, which is elevated in ac-CYB5R3–KO mice. Reactive oxygen and nitrogen species, including superoxide, hydrogen peroxide, and NO, can drive oxidation of heme and limit oxygen binding. Without a reduction enzyme present to counterbalance heme oxidation, oxygen delivery to mitochondria is restricted. Furthermore, oxidized heme can be displaced from protein, causing further oxidative stress and cellular damage. Based on our studies, it is likely that CYB5R3 serves this critical role to reduce oxidative stress not only via CoQ redox regulation, but also through its ability to reduce heme in metmyoglobin and sGC, so that optimal oxygen and NO binding can occur for maintenance of oxidative phosphorylation, mitochondrial health, and ATP levels in healthy cardiomyocytes. Future studies aimed at boosting redox balance with drugs, such as the FDA-approved Nrf-2 activators (i.e., dimethyl fumarate) and mitochondria-targeted redox therapies (i.e., MitoQ) may help define critical steps that initiate, propagate, and amplify the redox imbalance in ac-CYB5R3–KO mice.

An important observation gleaned from this study is that female mice with cardiomyocyte deletion of CYB5R3 do not exhibit the same phenotype as males. Recent advances in the field have noted important sex differences in the cardiovascular system, particularly regarding heart disease. It is known that premenopausal women have reduced incidence for coronary heart disease compared with age-matched men (59, 60). Preclinical animal models also show female-specific cardioprotection and reduced susceptibility to ischemic heart injury (61–63). Emerging studies have uncovered an important role for redox signaling, in particular aldehyde and NO signaling, for cardioprotection in females (64). For example, *S*-nitrosoglutathione reductase, an enzyme that regulates *S*-nitrosothiol signaling, is higher in female mice relative to males (65, 66). A similar sex difference for higher NO synthase expression in female mice versus males has also been shown (61, 67, 68). These known sex differences may explain why female CYB5R3-KO mice do not exhibit the same hypertrophy and SCD phenotype as males. Future studies focused on modulating NO and aldehyde signaling in male CYB5R3-KO mice are warranted.

Finally, we discovered that the high-frequency missense genetic variant rs1800457, which translates into a CYB5R3 T117S mutation with partial loss of function in cardiomyocytes, associates with decreased event-free survival (~20%) in Black persons suffering from HFrEF. It is important to note that the Black population has a higher incidence of out-of-hospital SCD when compared with the White population (69). In these patients, progressive HFrEF can lead to cardiac electrical instability and fatal arrhythmia, particularly when they have advanced disease (49). Notably, improved survival in Black patients only occurs with combination therapy that includes FDC I/H and an NO donor/antioxidant on top of standard therapy (41), suggesting that redox imbalance is impaired in this patient population. Unfortunately, FDC I/H is prescribed to fewer than 25% of Black patients who would potentially benefit (70). Several treatment barriers also exist, including (a) patient compliance with the existing drug dosing regimen (2 tablets, 3 times daily) and (b) drug side effects, such as headaches and dizziness. Identifying CYB5R3 T117S as having reduced activity (~50%) and associating with reduced event-free survival in Black patients with HFrEF suggests that the T117S genetic variant may be a contributor to SCD in persons of African ancestry with HFrEF. Thus, a multifront push that includes repurposing redox-targeting therapies, understanding the impact of genetic modifiers, and development of new precision medicines and strategies is greatly needed for identifying individuals at high risk for HFrEF and SCD to improve the timing and choices of therapeutic interventions that can improve their health.

### Study limitations.

There are two limitations to this study. The first limitation is that CYB5R3 is known to govern multiple cellular functions, including heme and CoQ reduction, elongation and desaturation of fatty acids, cholesterol biosynthesis, and drug metabolism. Because of this, narrowing down pathway hierarchy to the observed phenotype presents a major challenge. However, future endeavors geared toward modulating components of specific downstream pathways, such as cGMP, may provide valuable insight for overcoming this challenge. The second limitation in this study pertains to the relevance of CYB5R3 expression to clinical pathophysiology. Data from our TAC studies show that progression to HF may include an upregulation of CYB5R3 expression, possibly as an age-associated compensatory response that mechanistically factors into cardiomyocyte remodeling. Nonetheless, predisposition to HF and SCD occurs when ac-CYB5R3 is lost. In humans, the T117S partial loss-of-function variant is associated with poor cardiac outcomes. From a translational perspective, these data may suggest that reduced expression or activity of CYB5R3 in the presence of cardiovascular stressors may render subsets of individuals more susceptible to HF and SCD. So that we can better understand the clinical pathophysiology of CYB5R3 in HF, future studies should investigate whether the T117S variant in mice phenocopies T117S in human carriers. In summary, our study demonstrates essential roles of CYB5R3 in cardiomyocyte redox biology and identifies a genetic biomarker in individuals of African descent that may portend an increased risk of death from HFrEF.

## Methods

See Supplemental Methods for extended methods and materials

### Generation of a cardiomyocyte-specific CYB5R3 mouse.

Female *Cyb5r3* floxed mice (*Cyb5r3^fl/fl^)* (as previously described; ref. 27) were crossed with male α-myosin heavy chain/myosin heavy chain 6 promoter-driven tamoxifen-inducible cre-recombinase mice (B6.FVB[129]-A1cf^Tg(Myh6-cre/Esr1*)1Jmk^/J, Jackson Laboratories; short-hand, *Myh6-Cre*ER^T2+/–^). Heterozygous *Cyb5r3^fl/WT^*, *Myh6-Cre*ER^T2+/–^ littermates were crossed to produce heterozygous cre-positive, CYB5R3 homozygous WT control (*Cyb5r3^WT/WT^* *Myh6-Cre*ER^T2+/–^) and heterozygous cre-positive tamoxifen-inducible CYB5R3 homozygous KO (*Cyb5r3^fl/fl^ Myh6-Cre*ER^T2+/–^) animals. Mice were genotyped using primers described in Supplemental Table 1. Animals were originally of FVB/B6 mixed background and were backcrossed 3 times into the C57BL/6J background prior to use. Animals were aged 10 to 13 weeks before initiating intraperitoneal tamoxifen (33 mg/kg/d for 5 consecutive days) injections, which in *Cyb5r3^fl/fl^Myh6*-*Cre*ER^T2+/–^ mice produced cardiomyocyte-specific CYB5R3-KO (*Cyb5r3^Δ/Δ^Myh6-Cre*ER^T2^). Animals were euthanized by CO_2_ asphyxiation and hearts excised for calculation of total heart weight (including atria) to total body weight ratio. CYB5R3-KO efficiency was confirmed via qRT-PCR, Western blot, and RNA-Seq.

### RNA isolation, reverse transcription, and quantification.

Frozen heart tissue (30–50 mg) was pulverized over liquid nitrogen using a mortar and pestle. RNA was isolated from powdered tissue using the QIAGEN RNeasy Mini Kit (QIAGEN, 74106). RT was then performed with 1 μg RNA using Superscript III Reverse Transcriptase (Thermo, 18080093) to create a cDNA library. *Cyb5r3* and *Pgc1* expression were assessed via qRT-PCR using SYBR Green PCR Master Mix (Thermo, 4344463) reagents on the QuantStudio5 Real-Time PCR System. cDNA expression was normalized to the housekeeping gene glyceraldehyde 3-phosphate dehydrogenase (*Gapdh*). Primer sets are shown in Supplemental Table 1. Relative expression levels are shown as log fold change relative to *Gapdh*, with triplicate Ct values averaged for each sample.

### RNA-Seq analysis.

RNA-Seq was performed using core services at the Rangos Research Center. RNA was purified with the RNeasy MinElute Cleanup Kit (QIAGEN, 74204), and each sample was assessed for quantity and quality using a Qubit 2.0 fluorometer (Thermo Fisher) and Agilent Bioanalyzer Tape Station 2200. Library preparation was done using the Illumina TruSeq Stranded Total RNA Sample Prep Kit. cDNA libraries were quantitated and validated for quality similar to that of the purified RNA prior to being pooled (1.8 pM final concentration). Cluster generation and 75 bp unpaired read sequencing were performed on an Illumina NextSeq500. Sequencing analysis was performed using RNA-Seq on a Maverix Analytic Platform (Maverix Biomics Inc.). Quality control for raw fastq files was performed with FastQC^3^. Adapter sequences and primers were trimmed using fastq-mcf of ea-utils and Trimmomatic. Reads were mapped to the mouse genome (mm9) using STAR. Fragments per kilobase of transcript per million mapped reads (FPKM) for each gene were determined by Cufflinks and used as input for pairwise differential expression quantified by Cuffdiff. Read counts were then normalized across all samples, and significant differentially expressed genes were determined by adjusted *P* value with a threshold of 0.05. The data discussed in this publication were deposited in NCBI’s Gene Expression Omnibus database (GEO GSE206121).

### Protein analysis and quantification.

Homogenized frozen mouse heart tissue (30–50 mg) was lysed in 1× Cell Lysis Buffer (Cell Signaling, 9803S) containing protease (MilliporeSigma, P8340) and phosphatase inhibitors (MilliporeSigma, P5726). Lysates were sonicated and quantified using BCA protein assay (Thermo, 23225). Sample lysates containing 15 g of protein in 1× Laemmli buffer were prepared by boiling at 100°C for 10 minutes. Samples were loaded into 4%–12% Bis-Tris SDS Gels (Life Tech, NP0321BOX) and underwent electrophoresis at 140V for 90 minutes completed with MES Running Buffer. Proteins were transferred to nitrocellulose membranes in Tris-Glycine buffer for 1 hour at 100V. Membranes were blocked in LI-COR Odyssey Blocking Buffer (LI-COR 927-40003) diluted 1:1 with PBS (pH 7.4), followed by blotting overnight at 4°C with primary antibody in 1:1 LI-COR Odyssey Blocking Buffer/PBST (0.1% Tween PBS pH 7.4). Blotting was performed with LI-COR secondary antibodies for 1 hour at room temperature, followed by several washes with PBST and signal detection with a LI-COR Odyssey imaging system. Integrated intensities were quantified using Image Studio Lite (LI-COR), and protein expression was normalized to either β-actin (1:5000, Santa Cruz Biotechnology Inc., sc-47778), α-tubulin (1:5000, MilliporeSigma, T9026), TOM-20 (1:1000, Santa Cruz Biotechnology Inc., sc-11415), or GAPDH (1:1,000, Novus, NB300-221). Supplemental Table 2 details antibodies and concentrations used. Supplemental Figures 9–12 show full, uncropped Western blots, and blue dotted and solid boxes show cropped areas in main and supplemental figures.

### T117S genotyping in African American HF cohort.

gDNA was isolated from whole heart tissue using the PureLink Genomic DNA Mini Kit (Invitrogen, K1820-01). gDNA was quantified via NanoDrop (Thermo Fisher). For participants in the GRACE and GRAHF studies, DNA was isolated from peripheral blood by leukocyte centrifugation and cell lysis using the PureGene DNA Purification Kit (Gentra Systems). DNA (2 ng) was loaded into a 384-well plate with TaqMan Genotyping Master Mix (Thermo, 4371353) and CYB5R3 rs1800457 SNP Genotyping Assay (Thermo, 2986292_20). Analysis distinguished genotypes based on VIC/FAM fluorescence amplification (Thermo Fisher).

### Outcomes analysis.

For analysis of the impact of the T117S genotype on clinical outcomes in chronic HFrEF, participants from 2 prospective genetic outcomes studies were combined. The GRAHF cohort was a genetic substudy of the AHeFT trial (ClinicalTrials.gov NCT00047775) (41, 42). AHeFT was a placebo-controlled randomized trial of FDC I/H specifically in self-identified African Americans with chronic HFrEF. The GRACE cohort was a single-center genetic outcomes registry based at the HF clinic at the University of Pittsburgh Medical Center (43, 44). For the combined cohort, subjects treated with FDC I/H were excluded. Inclusion criteria were (a) chronic HFrEF with an LV EF of less than 0.45; (b) self-designated race of Black or African American; (c) New York Heart Association (NYHA) functional class of I–IV (https://www.heart.org/en/health-topics/heart-failure/what-is-heart-failure/classes-of-heart-failure). Subjects in the combined cohort were followed to an outcome of death, cardiac transplantation, or HF hospitalization. Event-free survival was compared by T117S genotype.

### Statistics.

GraphPad Prism Software, version 7.0d, was used for graphing and statistical analysis. Normality was determined using D’Agostino and Pearson’s normality test. Significance was determined by an unpaired, 2-tailed Student’s *t* test, 1-way ANOVA with Tukey’s multiple comparisons test, or χ^2^ test. *P* < 0.05 was considered statistically significant. Welch’s correction was used when SDs were dissimilar. Mann-Whitney *U* test was used for data not normally distributed. Error bars represent SEM. Each independent sample is overlaid on bar graphs to show spread and distribution of samples. Significance for event-free survival in human HFrEF analysis was determined using a log-rank test.

### Study approval.

Consent to use of human DNA was approved under University of Pittsburgh Institutional Review Board protocols (IRB0404033, IRB0401173, and IRB0504171) and was obtained as part of the main written consent form. Animal studies were approved and conducted under the University of Pittsburgh Institutional Animal Care and Use Committee (protocol number 19116317).

## Author contributions

NTC and ACS conceived and designed all of the experiments. SAH generated and managed CYB5R3 KOs and control mouse lines and conducted telemetry and TAC surgeries. HMS and HMA performed and analyzed histology. KCW performed complete blood count analyses. BG and GS performed optical mapping. MT provided interpretation of calcium measurements. SY performed RNA-Seq analysis. PN performed quinone measurements. PHT generated recombinant protein. MPM, RH, and JCG performed enzyme activity studies. MF and FJS measured lipids. YLW, MCS, and SH conducted DTI measurements, and BAK measured mtDNA/gDNA. CFM provided human VAD samples, and DMM provided human samples and analysis from the GRAHF and GRACE cohorts. NTC performed all other studies. NTC and ACS prepared figures and drafted the manuscript. All authors edited the manuscript.

## Supplementary Material

Supplemental data

## Version 1 09/15/2022

Electronic publication
