# Supplementary material for: Loss of cardiomyocyte CYB5R3 impairs redox equilibrium and causes sudden cardiac death
Source: J Clin Invest. 2022 Sep 15;132(18):e147120. doi: 10.1172/JCI147120 (PMC9479700; doi:10.1172/JCI147120)
Supplement: Supplemental data [file jci-132-147120-s089.pdf]

Extended Methods

Transverse aortic constriction and echocardiography

Male C57Bl/6 mice (Jackson Labs), between the age of 10-12 weeks, were anesthetized with 2.5% isoflurane and oxygen. The surgical area was prepared and a horizontal skin incision 0.5–1.0 cm in length was made at the level of the suprasternal notch. The thyroid was retracted, and a 2 to 3mm longitudinal cut made in the proximal portion of the sternum. Ligatures equivalent to a 28-guage needle were placed around the aortic arch. The skin was closed using a continuous suture pattern. Mice were allowed to recover and characterized at 7, 14 or 27 days post-surgery via echocardiography as previously described. (1)

Lipid analysis

Solvents used for extractions and mass spectrometric analyses were from Burdick and Jackson (Muskegon, MI). Heart samples (≈ 15-100 mg) were homogenized in a FastPrep-24™ 5G blender in 0.5 ml phosphate buffer 50 mM pH 7.4 in the presence of 1 mg butylated hydroxytoluene (BHT). An aliquot of the homogenate (200 µl) was diluted with 300 µl of the same buffer and lipids extracted using the Bligh and Dyer method (2). The organic extracts were dried under N_2 flow, resuspended in 200 µl methanol, and further diluted 100-fold in the same solvent. Lysophosphatidylcholines (Lyso-PCs) and phosphatidylcholines (PCs) were analyzed by high-performance liquid chromatography-tandem mass spectrometry (HPLC-MS/MS) using an analytical C18 Luna column (2 × 100 mm, 5 µm, Phenomenex) at a 0.3 ml/min flow rate, with a gradient solvent system consisting of 20 mM ammonium acetate (NH_4Ac) in water (solvent A) and 20 mM NH_4Ac in methanol (solvent B). Samples were chromatographically resolved using the following gradient program: 10-90 % solvent B (0.1-3 min); 90-100 % (3.0-3.1 min), 100% solvent B (3.1-13 min), followed by 2 min re-equilibration to initial conditions. Analysis was performed on a QTRAP 6500+ triple quadrupole mass spectrometer (AB Sciex, Framingham, MA) using the following parameters in positive ion mode: declustering potential (DP) 80 V, entrance potential

(EP) 15 V, collision energy 50 eV, collision cell exit potential (CXP) 15 V, and source temperature 500 ºC. Multiple reaction monitoring (MRM) transitions for Lyso-PCs and PCs were used following the charged loss of the characteristic phosphocholine head group ion (*m/z* 184). Results were reported as peak area/ mg of tissue.

Hematological analysis

Blood (5% v/v EDTA) was collected via left heart puncture in isoflurane-anesthetized mice and subjected to complete blood count analysis using a HemaVet 950 (Drew Scientific Inc, Miami Lakes FL).

Telemetry

Mice were anesthetized with isoflurane, and telemetry units (HDX-11, Data Sciences International [DSI]) were surgically implanted with leads placed on either side of the chest. Mice were allowed to recover for 2 weeks post-surgery before telemetry units were turned on. ECG recordings and heart rate were then measured continuously to assess baseline physiological BP. ECG recordings and heart rate measurements were recorded by Ponemah Software (DSI) and exported into Microsoft Excel (version 16.16.6) for analysis.

Open chest hemodynamics

Mice were anesthetized using inhaled isoflurane and ventilated via tracheostomy with positive pressure ventilation. Maintenance of anesthesia during the procedure is achieved using 3% isoflurane gas and between 21% and 100% oxygen delivered via inhalation tube. The mouse was intubated and placed in a supine position for longitudinal dissection of the sternum to expose the heart and great blood vessels. The left ventricle was cannulated with a 1.5F micro pressure-volume catheter (Transonic FTH-1211B-0018). The right ventricle was also catheterized, using real-time pressure tracings to identify the location for measurement of RV systolic pressure. Both catheters were secured in place with ligation sutures for recording 10 minutes of LVSP and RVSP,

heart rate, stroke volume, and cardiac output. A miniature ultrasound flow meter/probe placed at the level of the pulmonary artery and ascending aorta provided Doppler ultrasound spectrographic data for computation and calculation by the Pulsed Doppler Ultrasound Digital Signal Processor Workstation (Indus Instruments).

Optical mapping of calcium transience in perfused hearts

Mice were euthanized with euthasol, the heart dissected and immediately cannulated (>60 s) at the aorta, perfused with physiological Tyrode's solution (37°C, pH 7.4; 112 mM NaCl, 25 mM $NaHCO_3$, 5 mM KCl, 1.8 mM $CaCl_2$, 1.2 mM $MgSO_4$, 1 mM KH_2PO_4, 50 mM D-glucose; bubbled with 95% O_2 and 5% CO_2) and mounted on a custom-designed chamber for optical mapping in a Langendorff apparatus. The fluorescent calcium-indicator (40 µl of 0.5 mg/ml Rhod-2/AM in DMSO) was added as a bolus injection in the bubble trap proximal to the cannula, which served as a compliance chamber. Light from a 100-W tungsten lamp was collimated, passed through a 530 ± 30 nm interference filter, reflected by a 500 nm dichroic mirror and focused on the heart with a Nikon camera lens. To measure cytosolic free Ca^{2+}, fluorescent light from the heart was collected by the same lens, split by a 620 nm dichroic mirror to focus heart images at wavelengths of 570-595 nm on a CMOS camera (100 x 100 pixel, UltimaOne, Scimedia, Ltd. Tokyo Japan). Each camera scanned at 1,000 frames/s for 5 ms scan time, using an optical magnification to yield a spatial resolution of 150x150 μm^2 per pixel. Arrhythmia vulnerability was tested by varying the rate of stimulation from the basic sinus rhythm rate (typically 200 ms cycle length (CL)) to the fastest rhythm rate tolerated by the heart (typically 75 ms CL). Programmed stimulation was also used to test arrhythmia vulnerability by applying 10 impulses on the RV at S1-S1 intervals of 200 ms CL, followed by a premature impulse S2, then S1-S2 decreases by 10ms steps while S1-S2>100 ms, then decreasing S1-S2 by 2ms steps until failure of the heart to capture (refractory period) or S2 triggered arrhythmia (3-5).

Transmission electron microscopy

Heart tissue was fixed for 10 min in glutaraldehyde (2.5% in 0.1 M PBS, pH 7.4) and then cut into 1 mm^3 blocks for additional fixing (50 min). Tissue was washed (3 x 15 min) in PBS (0.1 M, pH 7.4) and then soaked in osmium tetroxide (1%) containing 1% potassium ferricyanide for 1 hr. Wash cycles were repeated as before. Tissue was then dehydrated by a series of 15-min washes in increasingly concentrated ethanol in PBS: 30% twice, 70% once, 90% once, and 100% thrice. Ethanol-dehydrated samples underwent two 10-min washes in propylene oxide for additional dehydration. Tissue infiltration with a 1:1 mix of propylene oxide and epon (1-3 hr) was followed with pure epon (4°C overnight). Three more rounds of 1-hr tissue infiltration with epon occurred before tissue embedding in pure epon (37°C, 24 hr) and curing (60 ^{o}C, 48 hr). Sections were imaged at the University of Pittsburgh, School of Medicine Center for Biological Imaging on the JOEL JEM1011 (JOEL, Inc Peabody, MA) transmission electron microscope. Quantifications from TEM included crude counts of mitochondria and total mitochondrial area and consisted of averaging separate images (15,000x; n=3) for each sample (n=6-8). Mitochondrial count was done using the count function of ImageJ. Mitochondrial area was assessed as electron dense mitochondria using the “outline” and ‘set scale’ functions of ImageJ.

Heart histology

Fixed heart tissue was also stained with a Masson’s Trichrome Kit or with hematoxylin & eosin staining, as previously described. (6)

Mitochondrial-to-genomic DNA ratio quantification

Total DNA from whole heart tissue was prepared as described in (7). mtDNA/gDNA ratio compared $\Delta\Delta C_T$ of mouse *mt-nd1/TBP* ratios. Reactions of DNA, TaqMan Universal Master Mix (Applied Biosystems, Waltham, MA), and 5 μM primer (10 μl multiplex reactions in triplicate) were

performed with standard curve to ensure linearity of assay and test dilution effects on rations in StepOnePlus thermocycler as described in (8).

Quantification of CoQ redox state and abundance.

Detailed methods are described in Rodriguez-Aguilera *et al* (9). Briefly, hexane was used to extract lipids from heart homogenate (100 μL) (homogenate:hexane ratio 3:5 *v/v*). Samples were centrifuged (16,000 x g, 1 min, 4ºC) and 100 μL supernatant from each was immediately injected into a 166-126 HPLC system (Beckman-Coulter) equipped with an electrochemical detector (ECD, Coulochem III ESA). Separation was carried out in a 15 cm Kromasil C18 column (Scharlab, Spain) at 40ºC with a mobile phase of methanol/n-propanol (65:35) containing 1.42 mM lithium perchlorate (flow rate 1 ml/min). Oxidized and reduced CoQ electrochemical detector readings were used for quantification (9).

MRI sample preparation and acquisition

At the end point, following euthanasia of the mouse, the blood in the heart was flushed out by retrograde perfusion of phosphate-buffered saline (PBS) through the abdominal aorta for 2 minutes, followed by retrograde perfusion of 4% paraformaldehyde for 5 minutes for tissue fixation. The heart was then dissected out of the chest and fixed by immersion in 4% paraformaldehyde at 4°C for 2 days, followed by rehydration in PBS at 4°C for 2 days before transfer to 10% neutral buffered formalin. For MRI, the fixed mouse heart was removed from formalin and lightly blotted dry with paper towel to remove excess water on the heart surface. Each heart was then transferred to a 1.5 cc tube containing Fomblin-Y perfluoropolyether vacuum oil (Millipore Sigma, MW = 1800) to eliminate artifact susceptibility from tissue-to-air interface. *Ex vivo* diffusion tensor imaging (DTI) was carried out on a Bruker BioSpec 70/30 USR spectrometer (Bruker BioSpin MRI, Billerica, MA, USA) operating at 7-Tesla field strength, equipped with an actively shielded gradient system and a quadrature radio-frequency volume coil (inner-diameter 35 mm). DTI was acquired as follows: isotropic 117 µm resolution, 4 msec diffusion encoding

duration, 8 msec diffusion gradient separation, 30 diffusion sampling directions, b = 1044.64 s/mm^2, echo time (TE) = 16.525 msec, and repetition time (TR) = 800 msec. The diffusion MRI data were reconstructed using generalized q-sampling imaging (GQI) (10) with restricted diffusion quantification(11) provided by the DSI studio (https://dsi-studio.labsolver.org/, ver. 05/2020), with a diffusion sampling length ratio of 1.25, angular threshold 60^0, step size 0.021 mm, anisotropy threshold 0.0263, and total 1,000,000 tracts calculated.

***Ex vivo* ATP and cGMP assays**

Total ATP in mouse whole heart lysate was quantified via luciferase-based ATP Determination Kit (Thermo A2066). cGMP quantification was done via ELISA (Cell Signaling 4360) and was normalized to total protein quantified by BCA protein assay (Thermo 23225). Perfused, snap-frozen heart tissue (30-50 mg) was homogenized using mortar and pestle and lysed in 1x Cell Lysis Buffer (Cell Signaling 9803S) containing protease (Sigma P8340) and phosphatase (Sigma P5726) inhibitors plus1 μM 3-isobutyl-1-methylxanthine (IBMX, Sigma I5879).

Immunohistochemistry of oxidative stress and tunnel staining in mouse heart sections

Hearts were deparaffinized using two 5-min incubations in xylene, followed by serial incubations (2 x 5 min each) in decreasing concentrations of ethanol in distilled deionized water: 100%, 95%, 70%, and 0%. Antigen retrieval (H-3300, Vector Laboratories) was performed in the microwave for 20 min. Slides were washed with PBS, blocked for 1 hr in PBS containing 10% horse serum (Sigma H1270-100mL), and stained at 4°C overnight. Sections were stained with the following antibodies made up in PBS with 10% horse serum: 8-oxoguanine (1:50, Abcam ab206461), 4-hydroxynonenal (4-HNE) (1:100, EMD Millipore 393207), and AlexaFlour-647 conjugated wheat germ agglutinin (1:250, Thermo W32466). One section per slide was stained with rabbit and mouse IgG controls (I:1000 and I:2000, respectively: Vector Laboratories). Slides were washed with PBS, stained for 1 hr with goat anti-rabbit (1:1000, 594nm; Thermo R37117) and goat anti-mouse (1:1000, 488 nm; Thermo R37120) secondary antibodies in PBS with 10% horse serum.

Sections were washed twice with PBS and cover-slipped using ProLong Gold Antifade with DAPI (Invitrogen P36931). Heart sections were imaged using a Nikon A1 Confocal Laser Microscope (20x, 1096 x 1096 resolution). Z-stacks (1 µm increments) were taken with highest-intensity fluorescent point of focus set at ± 6 µm. Maximum intensity projections were created and ImageJ used to perform quantification in separate channels.

Hypoxyprobe staining of mouse heart sections

ac-WT and ac-CYB5R3 KO mice were injected intravenously with Hypoxyprobe™ (150 mg/kg i.v.; HP2-200Kit) and rested for 30-60 min before euthanasia. Upon euthanasia, hearts were perfused with PBS (pH 7.4), excised and fixed in paraformaldehyde (4% in PBS) for 24 hr. Hearts were subsequently transferred to 30% sucrose for ≥ 24 hr, followed by mounting in optimal cutting temperature compound (OCT) for longitudinal sectioning (12 µm) and staining. Extraction occurred by ice-cold acetone for 10 min, followed by washing in PBS for 5 min before blocking (1x fish skin gelatin and 1x bovine serum albumin in PBS) for 30-60 min. Sections were stained overnight with primary antibodies: Hypoxyprobe™ mAb-FITC (1:500, 488 nm, HP2-200Kit) and AlexaFluor conjugated wheat germ agglutinin (1:500, 647 nm, Thermo W32466). Tissues were mounted in ProLong Gold Antifade with DAPI (Invitrogen, P36931) and an Olympus Fluoview-1000 confocal laser microscope was used to acquire images (20x; 1096 x 1096 resolution). ImageJ was used for quantifying Hypoxyprobe-positive cells relative to total cells.

Generation of soluble CYB5R3 wildtype and CYB5R3 T117S recombinant protein

The Δ23 WT and Δ23 CYB5R3 T117S proteins were expressed as C-terminal fusions in the pET-Smt3 vector in *E. coli* BL21 (DE3) cells (Genlantis) (12). Donor cultures grown in LB at 37ºC overnight were diluted 1:50 into LB and grown at 37ºC until reaching an OD_{600} of 0.5-0.7. Expression cultures were induced with 1 mM IPTG and protein was expressed for 4-6 hours. Cells were harvested by centrifugation and lysed by sonication in 50 mM Tri-HCl, 150 mM NaCl, 10% v/v glycerol, pH 7.4 on ice. Soluble protein was separated by centrifugation (30,000 x g,

4ºC, 30 min) and the supernatant was applied to a NiNTA Fast Flow column (GE Lifesciences, USA) equilibrated with lysis buffer. The column was washed in lysis buffer supplemented with 60 mM imidazole and the protein was eluted in lysis buffer supplemented with 400 mM imidazole. Fractions containing the CYB5R3 protein were combined and concentrated using centrifugal concentrators (Amicon Ultra 10K, Millipore, USA). The His-tagged Smt3-specific protease, Ulp1, was added to cleave the fusion and the mixture was applied to a Sephacryl S-300 HR size exclusion column (GE Lifesciences, USA) equilibrated in lysis buffer. Fractions containing monodisperse protein were collected and subsequently reapplied to the NiNTA column to capture the His-tagged Smt3 and Ulp1 proteins. Flow-through containing the CYB5R3 protein was collected, and a final buffer exchange was performed using a Superdex S75 size exclusion column (GE Lifesciences, USA). Protein was concentrated, flash-frozen in liquid N_2 and stored at -80ºC prior to use.

CYB5R3 activity assay

Recombinant Δ23 CYB5R3 wildtype or Δ23 CYB5R3 T117S ± CYB5B were each diluted to 0.5 µM in 100 mM Tris-HCl pH 7.4, mixed together with 1 µM CYB5B, and pre-incubated at 37°C for 10 minutes. Isolated equine heart myoglobin (Sigma M1882) was solubilized in 100 mM Tris-HCl pH 7.4 at 33.3 µM, warmed to 37°C and 150 µL volume was loaded into each well of a clear flat bottom 96 well plate. A 100 µL volume of Δ23 CYB5R3 WT, or Δ23 CYB5R3 T117S ± CYB5B mix was added to the myoglobin solution and baseline absorbance spectra (450-700 nm) were obtained on a Biotek plate reader at 37°C. Myoglobin reduction was initiated by the addition of 10 uL of 2.5 mM NADH or 20 mM DTT and absorbance at 540 nm was read kinetically for 40 min at 37°C. Final reaction concentrations were as follows; 20 µM Mb, 0.2 µM Δ23 CYB5R3 wildtype or Δ23 CYB5R3 T117S, 0.4 µM CYB5B, 1 mM DTT, 100 µM NADH. Reduction rates were obtained by calculating the slope of the linear portion of the change in A 540 over time.

HEK 293FT cells (Thermo Fisher) that were stably knockdown with CYB5R3 (as previously described (13)) were grown to confluency and Lipofectamine 3000 (ThermoFisher L3000015) was used to transfect with cDNA for either the membrane bound or soluble version (Δ23) of WT and T117S CYB5R3 in pcDNA3.1. Cells were lysed in RIPA buffer containing protease inhibitors (Sigma PB340) and total protein quantified with Pierce™ BCA Protein Assay kit (ThermoFisher 23225). Overexpression lysates were diluted in 100 mM Tris-HCl pH 6.8, heated to 37 °C for 15 min and loaded into a clear bottom 96-well assay plate, 10 ug/well and 5 ug/well for membrane bound and soluble versions, respectively. 2,6-dichlorophenolindophenol (DCPIP, 150 uM) redox dye was added to the plate and baseline absorbance at 630 nm was taken on a Biotek plate reader. CYB5R3 reduction of DCPIP was catalyzed with the addition of 100 uM NADH and A_{630} was read continuously for 30 min at 37°C. Rate of reduction was calculated as change in A_{630} per second.

TUNEL Staining

Apoptosis was assessed with a DeadEnd™ Fluorometric TUNEL System (Promega G3250). Heart sections were deparaffinized using two 5-min incubations in xylene, a 5 min wash in 100% ethanol, followed by serial incubations (3 min each) in decreasing concentrations of ethanol in distilled deionized water: 100%, 95%, 70%, and 0%. Slides were then washed in 0.85% NaCL for 5 min and immersed in PBS for an additional 5 min. Sections were fixed by incubation in 4% paraformaldehyde in PBS for 15 min and washed 2x for 5 min in PBS. A 10 min incubation in 20 ug/mL Proteinase K solution permeabilized the sections, followed by a 5 min wash in PBS. This permeabilization step was repeated, flowed by a final wash before sections were treated with equilibration buffer and labeled by treatment with TdT reaction mix for 60 min at 37 °C in a humidified/light-tight chamber. Reaction was stopped by emersion in 2X SSC for 15 min followed by 3x 5-min washes in PBS. ProLong Gold Antifade mounting media with DAPI (Invitrogen P3693) was added to the slides, followed by imaging with a Nikon A1 Confocal Laser Microscope.

Maximum intensity projections were created and ImageJ was used to perform quantification in separate channels.

1. Durgin BG, Wood KC, Hahn SA, McMahon B, Baust JJ, and Straub AC. Smooth muscle cell CYB5R3 preserves cardiac and vascular function under chronic hypoxic stress. *Journal of molecular and cellular cardiology.* 2022;162:72-80.
2. Bligh EG, and Dyer WJ. A rapid method of total lipid extraction and purification. *Can J Biochem Physiol.* 1959;37(8):911-7.
3. Del Nido PJ, Glynn P, Buenaventura P, Salama G, and Koretsky AP. Fluorescence measurement of calcium transients in perfused rabbit heart using rhod 2. *Am J Physiol.* 1998;274(2):H728-41.
4. Baker LC, Wolk R, Choi BR, Watkins S, Plan P, Shah A, et al. Effects of mechanical uncouplers, diacetyl monoxime, and cytochalasin-D on the electrophysiology of perfused mouse hearts. *Am J Physiol Heart Circ Physiol.* 2004;287(4):H1771-9.
5. Salama G, and Hwang SM. Simultaneous optical mapping of intracellular free calcium and action potentials from Langendorff perfused hearts. *Curr Protoc Cytom.* 2009;Chapter 12:Unit 12 7.
6. Durgin BG, Hahn SA, Schmidt HM, Miller MP, Hafeez N, Mathar I, et al. Loss of smooth muscle CYB5R3 amplifies angiotensin II-induced hypertension by increasing sGC heme oxidation. *JCI Insight.* 2019;4(19).
7. Kolesar JE, Wang CY, Taguchi YV, Chou SH, and Kaufman BA. Two-dimensional intact mitochondrial DNA agarose electrophoresis reveals the structural complexity of the mammalian mitochondrial genome. *Nucleic acids research.* 2013;41(4):e58.
8. Falabella M, Kolesar JE, Wallace C, de Jesus D, Sun L, Taguchi YV, et al. G-quadruplex dynamics contribute to regulation of mitochondrial gene expression. *Sci Rep.* 2019;9(1):5605.
9. Rodríguez-Aguilera JC, Cortés AB, Fernández-Ayala DJM, and Navas P. Biochemical Assessment of Coenzyme Q(10) Deficiency. *J Clin Med.* 2017;6(3):27.
10. Yeh FC, Wedeen VJ, and Tseng WY. Generalized q-sampling imaging. *IEEE Trans Med Imaging.* 2010;29(9):1626-35.
11. Yeh FC, Liu L, Hitchens TK, and Wu YL. Mapping immune cell infiltration using restricted diffusion MRI. *Magn Reson Med.* 2017;77(2):603-12.
12. Mossessova E, and Lima CD. Ulp1-SUMO crystal structure and genetic analysis reveal conserved interactions and a regulatory element essential for cell growth in yeast. *Mol Cell.* 2000;5(5):865-76.
13. Yuan S, Hahn SA, Miller MP, Sanker S, Calderon MJ, Sullivan M, et al. Cooperation between CYB5R3 and NOX4 via coenzyme Q mitigates endothelial inflammation. *Redox Biol.* 2021;47:102166.

SUPPLEMENTAL TABLES

Supplemental Table 1. Primers used for genotyping and qRT-PCR.

	Gene	Forward (5'-3')	Reverse (5'-3')
**qRT-PCR**	*PPARGC1a*	TGTGTGCTGTGTGTCAGAGT	ACCAGAGCAGCACACTCTATG
	GAPDH	ATGACATCAAGAAGGTGGTG	CATACCAGGAAAATGAGCTTG
	Cyb5R3	CACCACTGTCTTCTTCGCCA	GAGGAGCGCTGAAACAGCTT
**Mouse Genotyping**	*A1cf*	AGCTTCTGTTCAGGCCATAG	GCGAATTCAGCATGCCACTT
	Cre	GCAAGAACCTGATGGACATGTTCAG	GCAATTTCGGCTATACGTAACAGGG
	Cyb5R3	GCAGCCTCAGGACTGTTTCT	TTGACCCTCTGCTGGAACTG

Supplemental Table 2. Antibodies and histological markers used for western blot and immunofluorescence.

Target	Vendor	Catalog # / Clone	Dilution
beta-actin	SantaCruz	sc-47778 / C4	1:5000
4-hydroxynonenal	Millipore	393207 / N/A	1:100
8-oxoguanine	Abcam	Ab206461 / 2Q23311	1:50
alpha-tubulin	Sigma Aldrich	T6074 / B-5-1-2	1:5000
CYB5R3	ProteinTech	10894-1-AP / N/A	1:5000
Heme Oxygenase-1	Abcam	ab68477 / EPR1390Y	1:5000
Mito Complex Cocktail	Abcam	ab110413	1:1000
CI	Abcam	ab110242 / 20E9DH10C12	
CII	Abcam	ab14714 / 21A11AE7	
CIII	Abcam	ab14745 / 13G12AF12BB11	
CIV	Abcam	ab14705 / 1D6E1A8	
CV	Abcam	Ab14748 / 15H4C4	
Myoglobin	Santa Cruz	sc-74525 / A-9	1:1000
phosphoVASP Ser239	Cell Signaling	3114 / N/A	1:1000
sGC-beta	Cayman Chemical	160897 / N/A	1:1000
TOM20	Santa Cruz	sc-11415 / FL-145	1:1000
VASP	Proteintech	13472-1-AP / N/A	1:1000
Wheat Germ Agglutinin	Thermo Fisher	W32466 / N/A	1:250
GAPDH	Novus	NB300-221 / 1D4	1:1000
LC3A	Cell Signaling	4599 / D50G8	1:1000

Supplemental Table 3. Top differential expression genes (*P*-value < 0.05) ranked by fold change.

ENSEMBL ID	Gene Name	log2FC	p-value
ENSMUSG00000095079	Igha	3.017046964	0.000328846
ENSMUSG00000067149	Jchain	2.75612313	5.09E-10
ENSMUSG00000027966	Col11a1	2.696708943	0.000105352
ENSMUSG00000076609	Igkc	2.498270548	3.84E-12
ENSMUSG00000086429	Gt(ROSA)26Sor	2.284213493	3.11E-51
ENSMUSG00000031849	Comp	2.175271563	2.78E-06
ENSMUSG00000016995	Matn4	2.066442102	9.33E-05
ENSMUSG00000079022	Col22a1	2.023348686	7.04E-05
ENSMUSG00000025889	Snca	1.770252832	0.018152464
ENSMUSG00000052305	Hbb-b1	1.733205589	0.020640701
ENSMUSG00000039058	Ak5	1.729124814	0.000336503
ENSMUSG00000073940	Hbb-bt	1.709938244	0.020574455
ENSMUSG00000031351	Zfp185	1.697881018	0.000889398
ENSMUSG00000074445	Sprr2a3	1.662346002	0.042716194
ENSMUSG00000078664	Sprr2a1	1.654739931	0.041533421
ENSMUSG00000068893	Sprr2a2	1.652649371	0.040811642
ENSMUSG00000026051	Ecrg4	1.641405996	0.010865266
ENSMUSG00000028626	Col9a2	1.600082684	0.00065369
ENSMUSG00000025270	Alas2	1.595044681	0.027699406
ENSMUSG00000069919	Hba-a1	1.591720988	0.037790216
ENSMUSG00000032942	Ucp3	-1.94464295	0.014830106
ENSMUSG00000025537	Phkg1	-2.015472558	8.48E-05
ENSMUSG00000030098	Grip2	-2.015485801	0.002453103
ENSMUSG00000059325	Hopx	-2.061513033	3.29E-07
ENSMUSG00000003545	Fosb	-2.070540023	0.004798794
ENSMUSG00000119584	Rn18s-rs5	-2.073536066	0.00097509
ENSMUSG00000028415	Spink4	-2.119808952	3.73E-05
ENSMUSG00000115610	Gm31251	-2.124233927	0.00642809
ENSMUSG00000002500	Rpl3l	-2.139736162	2.69E-06
ENSMUSG00000022032	Scara5	-2.204216356	2.76E-07
ENSMUSG00000053964	Lgals4	-2.320342921	0.002220001
ENSMUSG00000025784	Clec3b	-2.368966082	8.70E-17
ENSMUSG00000043085	Tmem82	-2.379635096	0.007492731
ENSMUSG00000003934	Efnb3	-2.386823697	1.43E-09
ENSMUSG00000090889	Gm17428	-2.390412735	0.004600595
ENSMUSG00000018554	Ybx2	-2.707975866	6.27E-05
ENSMUSG00000056973	Ces1d	-2.905136503	0.000108978
ENSMUSG00000021456	Fbp2	-3.113355522	5.81E-12
ENSMUSG00000019768	Esr1	-3.176248216	5.26E-05

ENSMUSG00000055632	Hmcn2	-3.461519839	6.30E-09

Supplemental Table 4. Baseline characteristics from a meta-analysis of the GRACE and GRAHF trials comparing AA CYB5R3 T117S non-carriers (CC) and carriers (CG/GG). *P*-value represents differences between CC and CG/GG.

Baseline Demographics	Overall	Non-carrier (CC)	T117S Carrier (CG/GG)	*P* value
N=	280	150	130	
age	55 ± 14	55 ± 14	55 ± 13	0.99
% female	39	38	40	0.81
% ischemic etiology	24	27	21	0.15
LVEF	0.30 ± 0.10	0.31 ± 0.10	0.30 ± 0.10	0.72
NYHA (% 1/2/3/4)	1/21/75/3	1/21/75/3	1/22/75/2	0.96
HR	74 ± 12	75 ± 12	73 ± 11	0.14
BP sys	123 ± 19	124 ± 17	123 ± 20	0.48
BP dia	76 ± 12	76 ± 11	75 ± 13	0.45
% ACE	78	81	74	0.20
% Beta Blockers	80	79	81	0.77
% Aldosterone Antagonist	32	31	32	0.90

SUPPLEMENTAL FIGURES

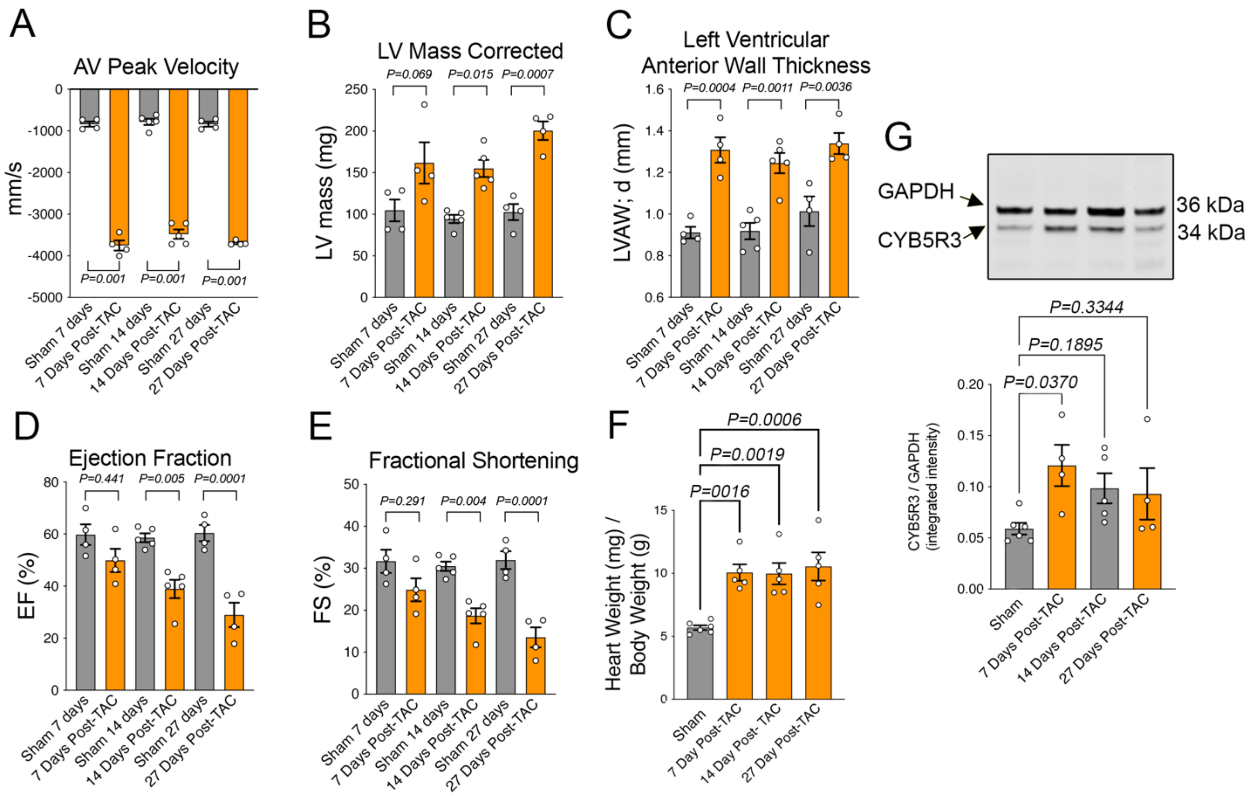

Supplemental Figure 1. TAC causes increased CYB5R3 expression. Echocardiography measurements of **A**) AV Peak Velocity, **B**) LV Mass Corrected, **C**) LV Anterior Wall Thickness, **D**) ejection fraction and **E**) fractional shortening with sham vs TAC compared at 7, 14 and 27 days. **F**) Heart weight to body weight ratio for sham vs TAC compared at 7, 14 and 27 days. **G**) Western blot of CYB5R3 and GAPDH for sham vs TAC compared at 7, 14 and 27 days with quantification shown below. Error bars show S.E.M, P-value calculated by a 1-way ANOVA.

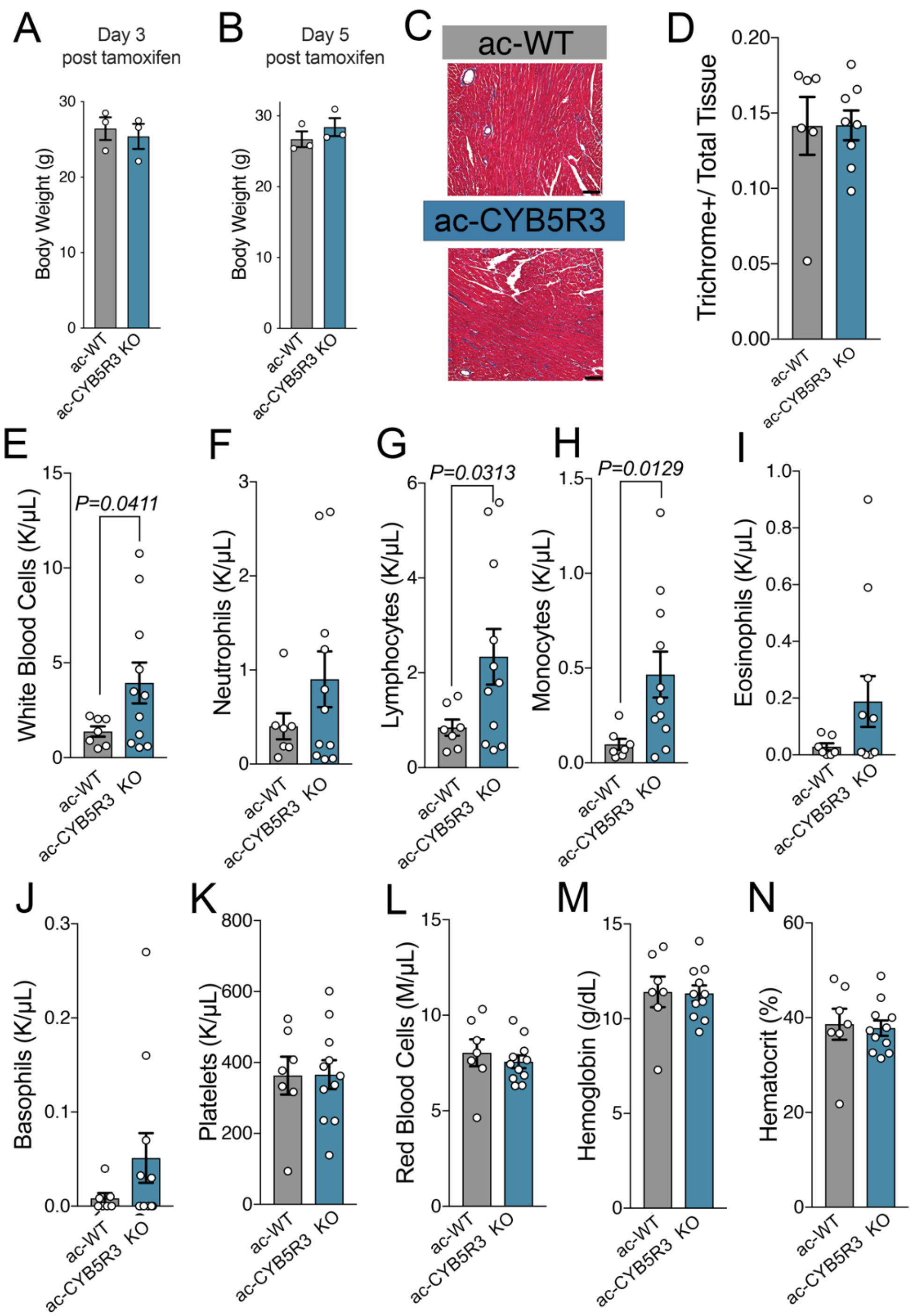

Supplemental Figure 2. Ac-CYB5R3 KO does not impact body weight or cardiac fibrosis but induces inflammatory response detectable via complete blood count. A-B) Body weight differences between ac-WT and ac-CYB5R3 KO mice on days 3 and 5 post-tamoxifen (n=3). **C**) Representative trichrome stained heart sections and **D**) quantification of trichome positive staining area relative to total tissue area (n=6-8). Hematology counts of circulating **E)** white blood cells, **F)** neutrophils, **G)** lymphocytes, **H)** monocytes, **I)** eosinophils, **J)** basophils, **K)** platelets, **L)** red blood cells, **M)** hemoglobin, and **N)** percent hematocrit (n=7-11). Error bars show S.E.M, P-value calculated by students t-test.

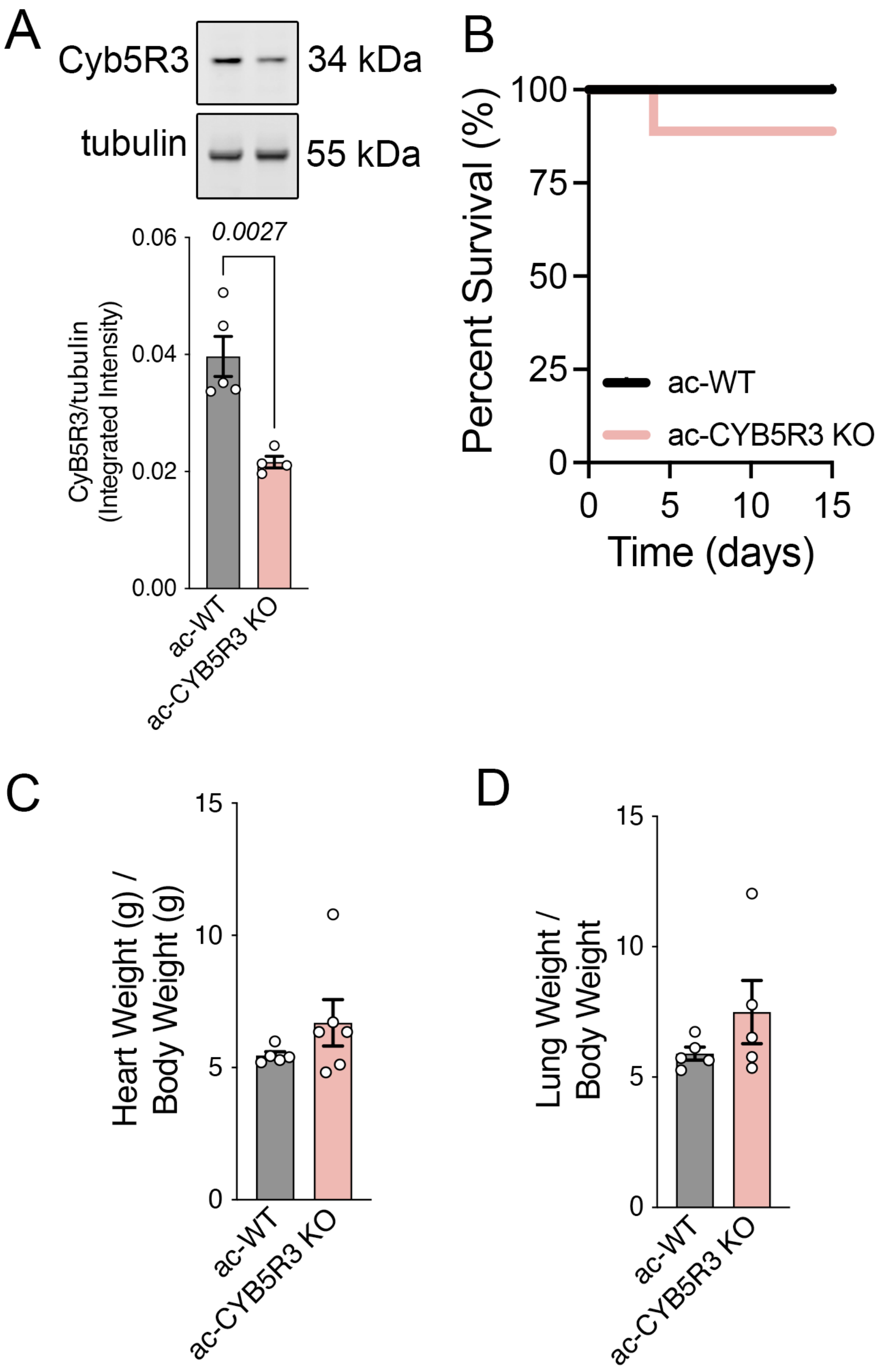

Supplemental Figure 3. ac-CYB5R3 KO in female mice does not cause SCD, hypertrophy or increased lung weight to body weight compared to ac-WT. A) Western blot analysis of total heart tissue from female ac-WT and ac-Cyb5R3 KO mice 5 days post-tamoxifen injection (n=4-5). **B)** Survival curve comparing female ac-WT (n=5) and ac-Cyb5R3 KO mice (n=4). **C**) Heart weight to body weight ratio (n=5-6) and **D**) Wet lung weight to body weight ratio (n=5) from female ac-WT and ac-Cyb5R3 KO. Error bars show S.E.M, P-value calculated by students t-test.

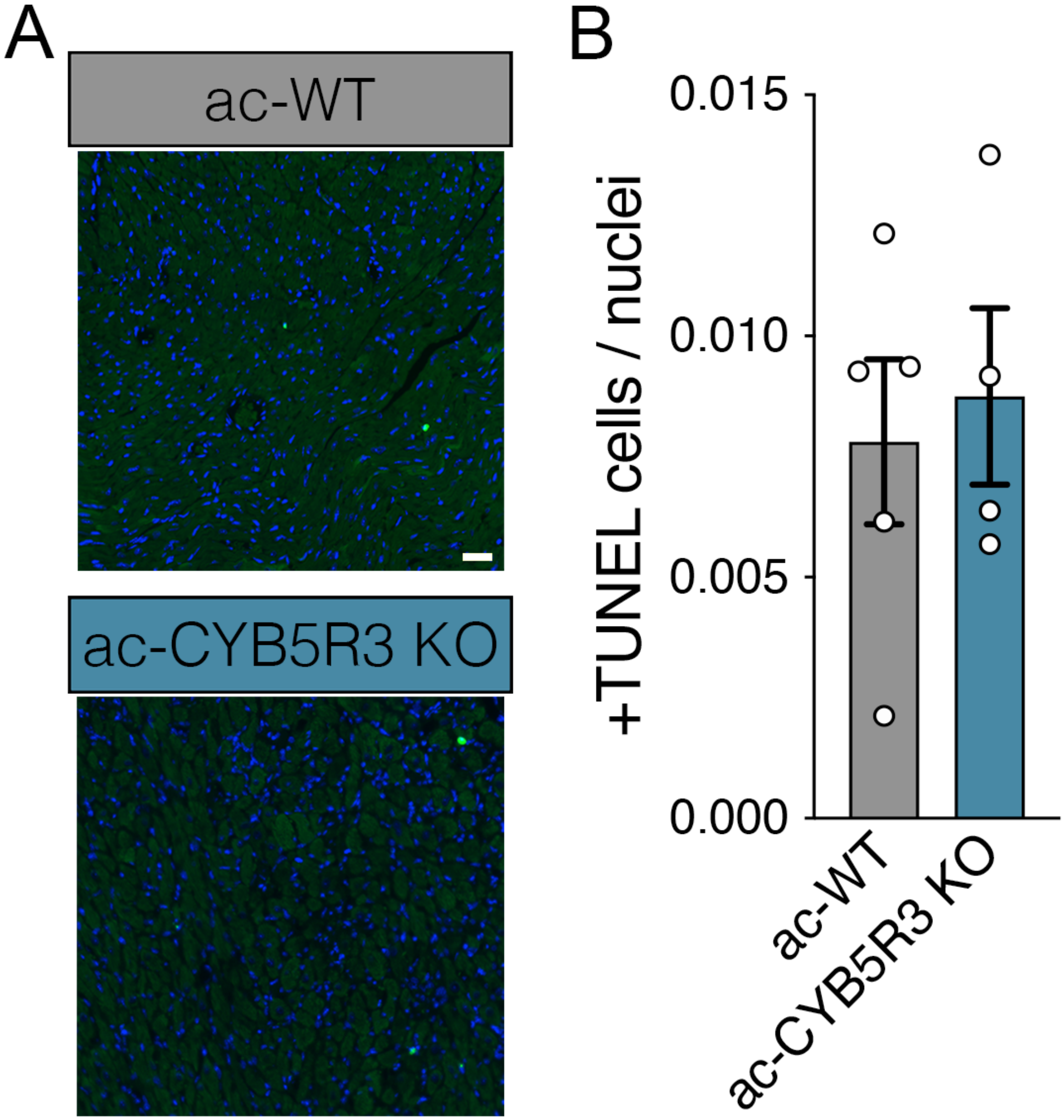

Supplemental Figure 4. ac-CYB5R3 KO does not cause increased apoptosis compared to ac-WT mice. A) Immunofluorescence images of +TUNEL cells from ac-WT and ac-CYB5R3 KO mice. **B**) Quantification of +TUNEL cell per nuclei (n=4-5). Error bars show S.E.M, P-value calculated by students t-test. Scale bar is 50 μm.

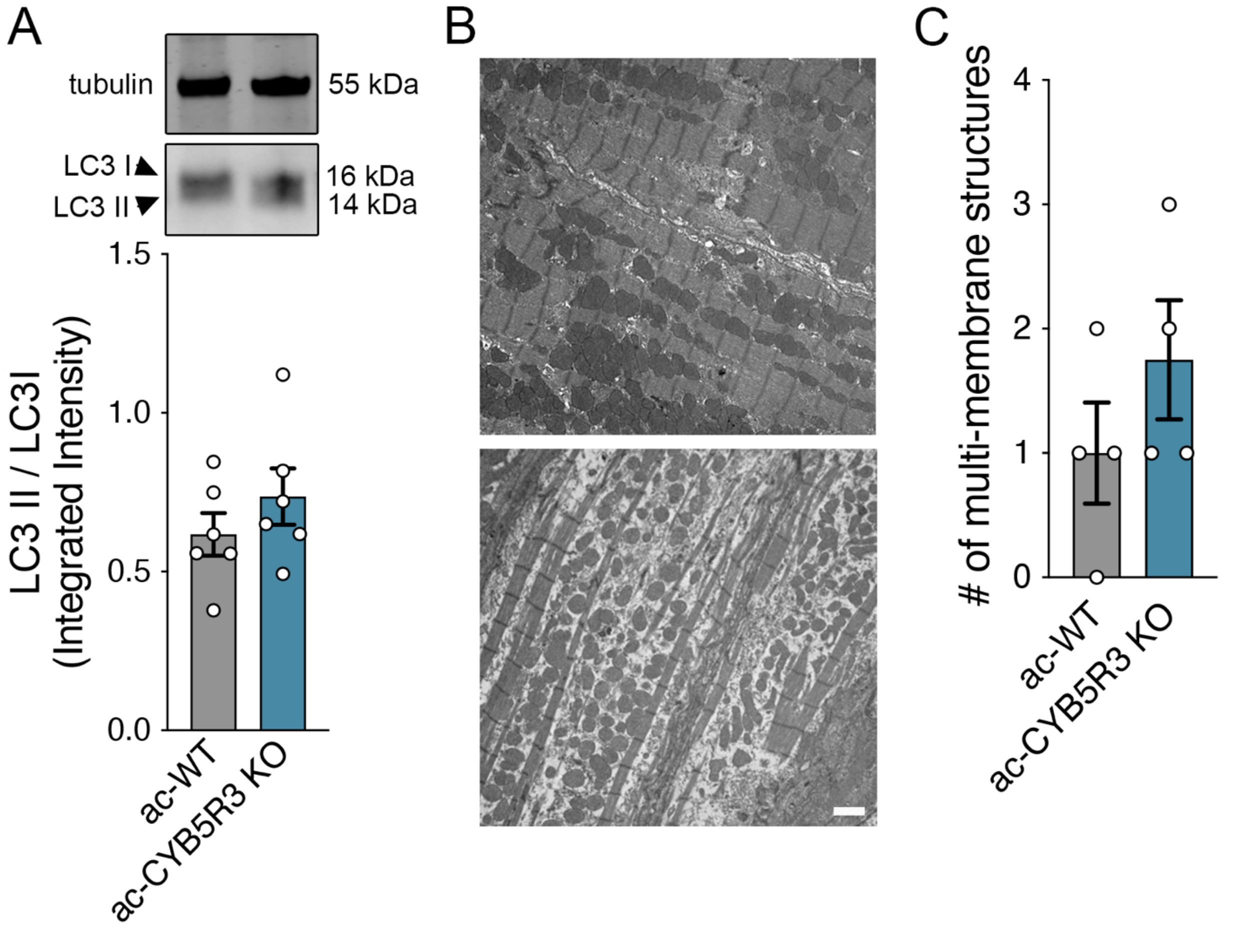

Supplemental Figure 5. ac-CYB5R3 KO does not impact autophagy measured by LC3I/LCII ratio or multi-membrane structures via TEM compared to ac-WT mice. **A**) Western blot of LC3 I/II and tubulin expression and quantification LC3 II/LC3 I ratio comparing ac-WT and ac-CYB5R3 KO in total heart tissue (n=6). **B**) TEM image of ac-WT (top) and ac-CYB5R3 KO (bottom) and **C**) quantification of multi-membrane structures from TEM images from ac-WT and ac-CYB5R3 KO hearts (n=4). Error bars show S.E.M, P-value calculated by students t-test. Scale bar is 1 μm.

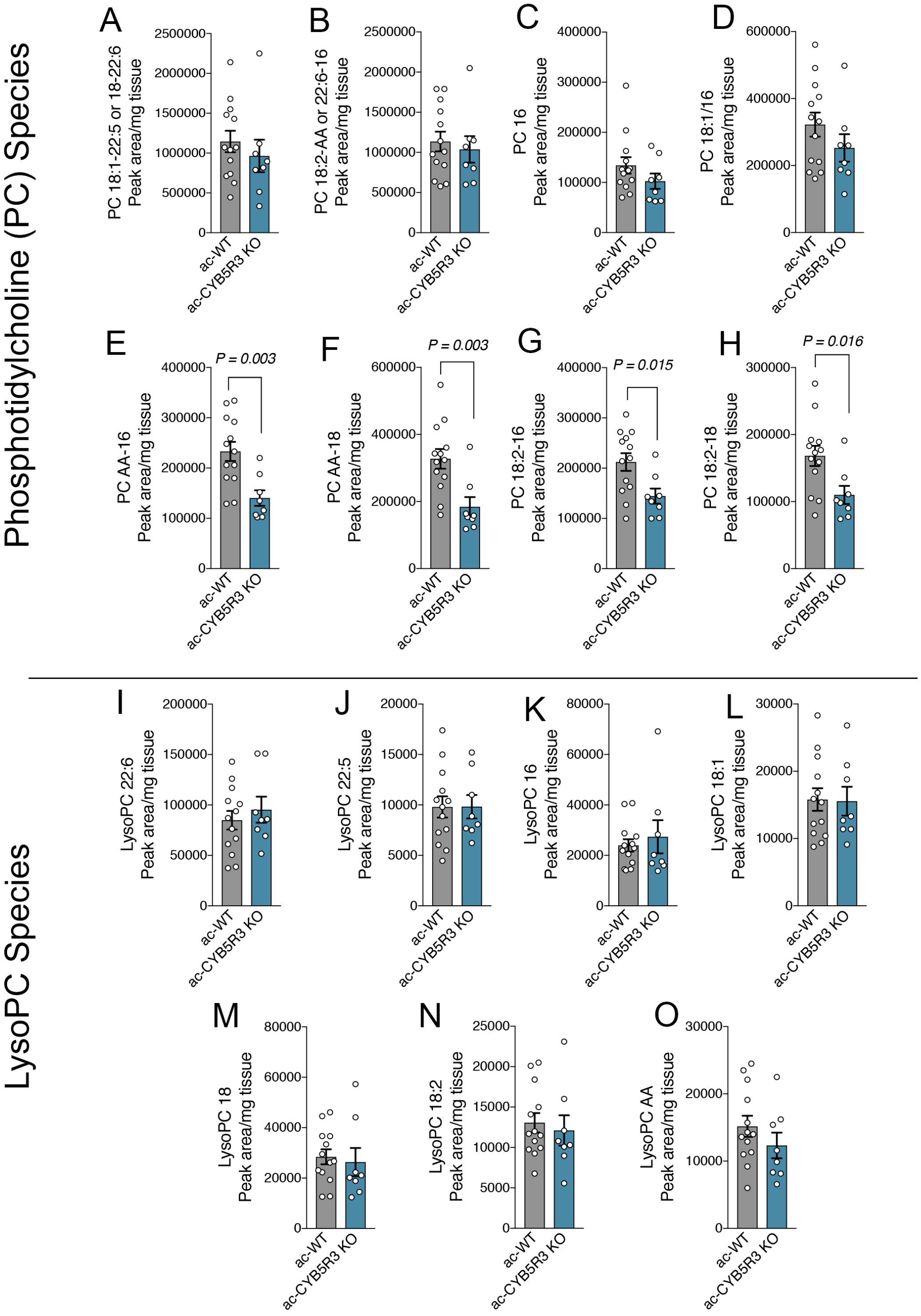

Supplemental Figure 6. Loss of cardiac CYB5R3 alters whole heart lipid profile. A-H) Relative quantitation of phosphatidylcholine (PC) species in cardiac tissue from ac-WT and ac-CYB5R3 KO mice (n=8-13). **I-N)** Relative quantitation of lysophosphatidylcholine (LysoPC) species in cardiac tissue from ac-WT and ac-CYB5R3 KO mice (n=8-13). Error bars show S.E.M, P-value calculated by students t-test.

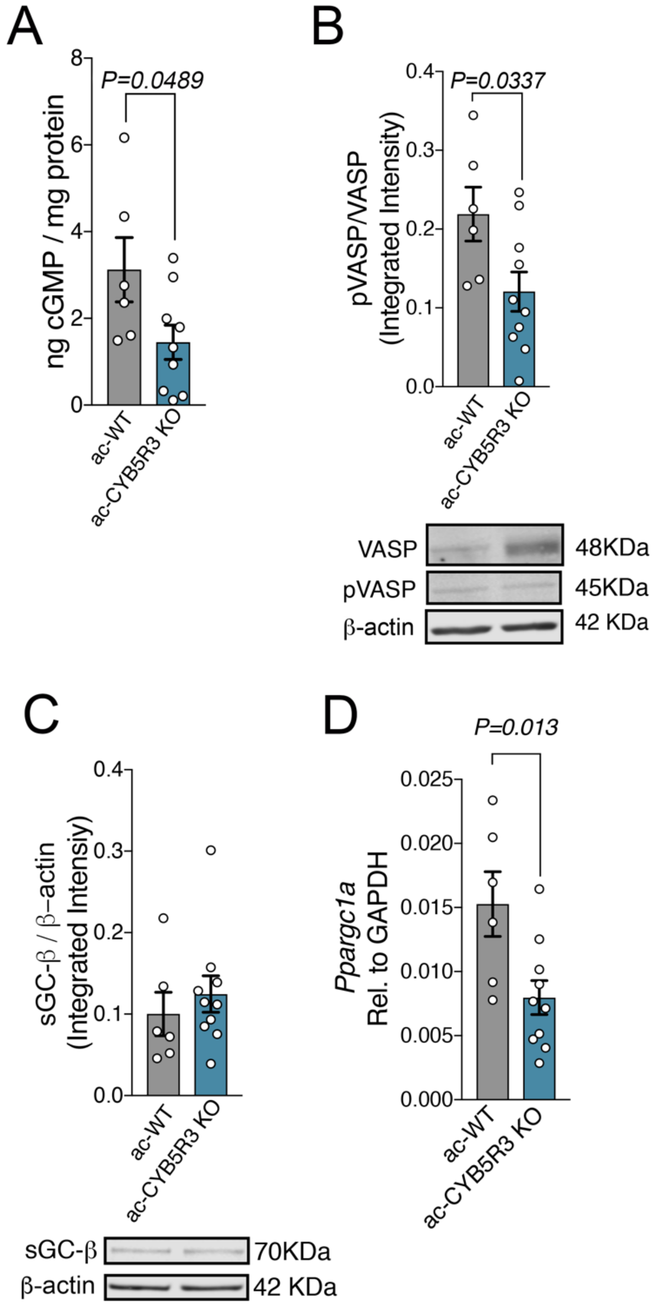

Supplemental Figure 7. Loss of Cardiac CYB5R3 reduces total cGMP and downstream signaling. A) cGMP measurements normalized to milligrams protein from ac-WT and ac-CYB5R3 KO mice (n=6-9). **B)** Quantification of integrated intensity ratio and representative images from Western blot of phosphorylated and total VASP, with β-Actin loading control (n=6-10). **C)** Western blot of representative bands from sGC-β and β-actin. Quantification of the integrated intensity of sGC-β relative to β-Actin loading control (n=6-10). **d**) *Ppargc1a* mRNA expression relative to GAPDH (n=6-10). Error bars show S.E.M, P-value calculated by students t-test.

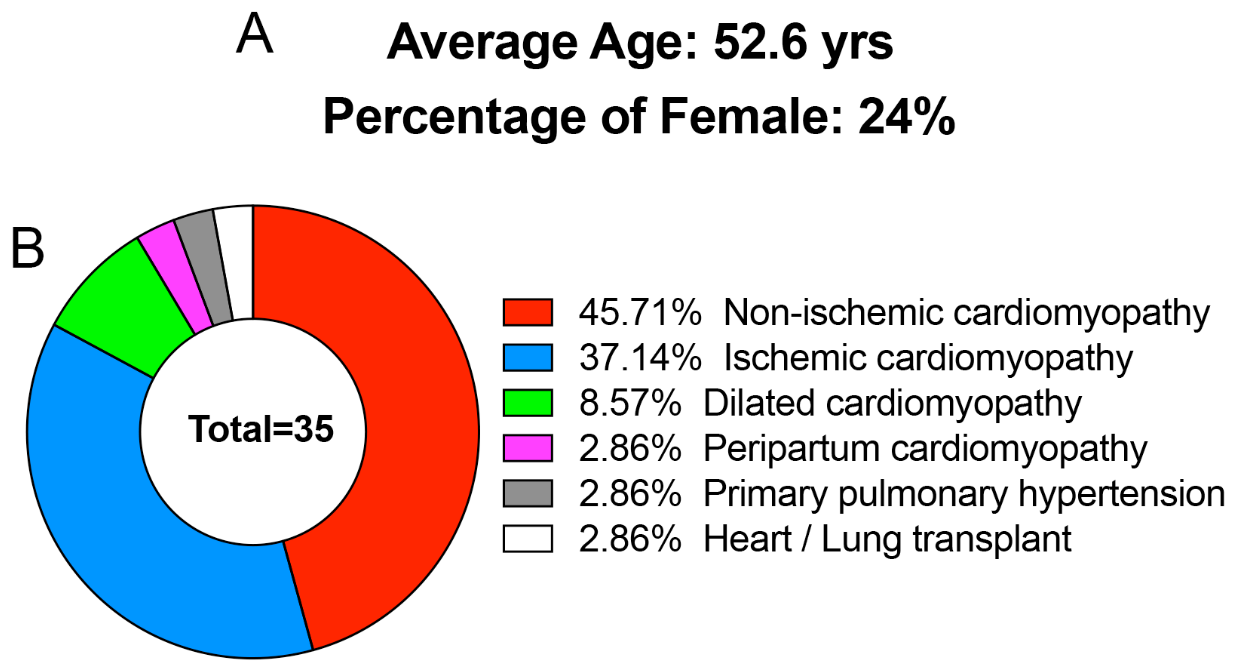

Supplemental Figure 8. Baseline characteristics of HF patients receiving transplant or LVAD.

Figure 1C uncropped WB

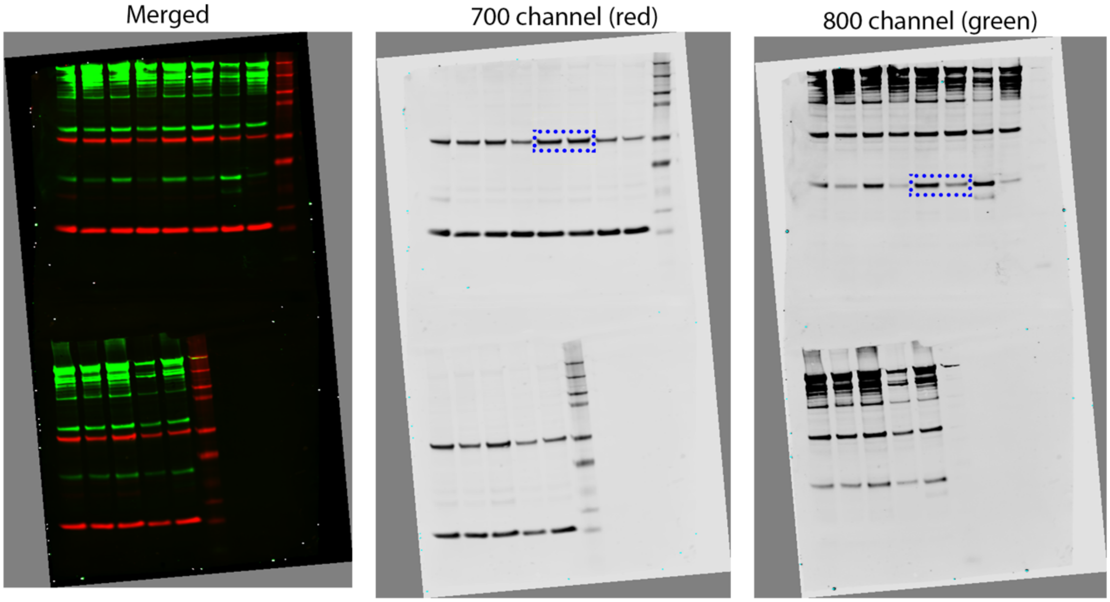

Figure 3H uncropped WB

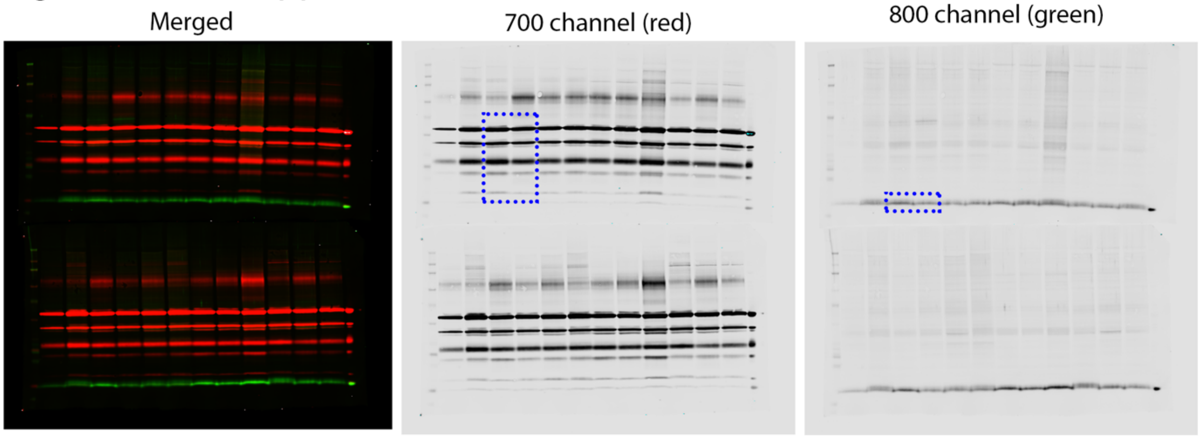

Supplemental Figure 9. Figure 1C uncropped Western blot for tubulin (700 channel, red) and CYB5R3 (800 channel, green). Figure 3H uncropped Western blot for mitochondria complex I-V (700 channel, red) and TOM20 (800 channel, green). Blue hashed box shows cropped area.

Figure 7E uncropped WB

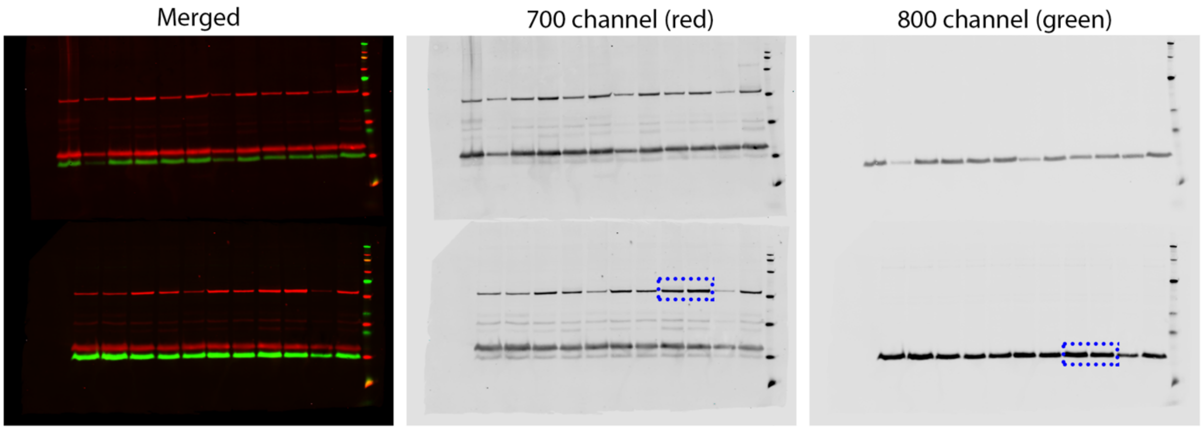

Figure 7F uncropped WB

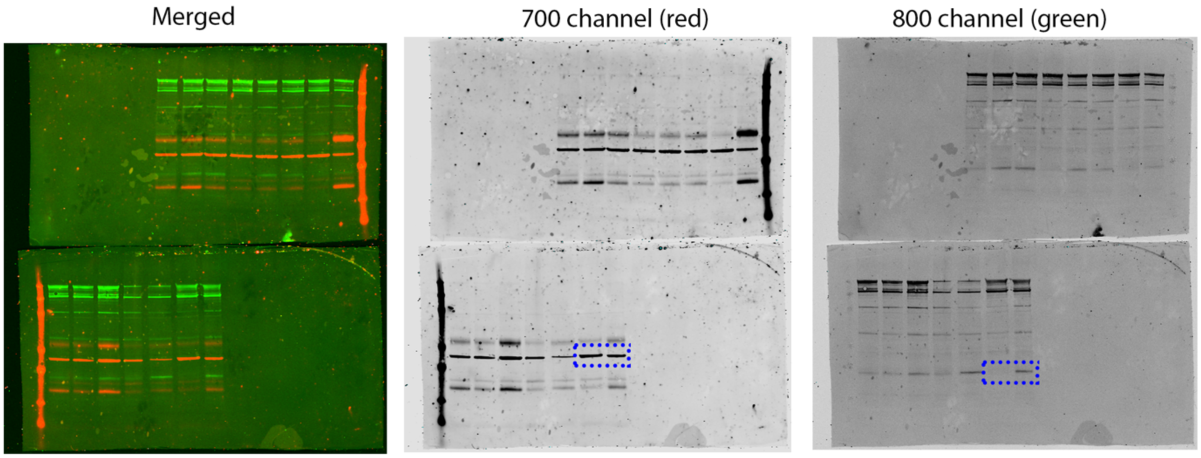

Supplemental Figure 10. Figure 7E uncropped Western blot for tubulin (700 channel, red) and myoglobin (800 channel, green). Figure 7F uncropped Western blot for β-actin (700 channel, red) and HO-1 (800 channel, green). Blue hashed box shows cropped area.

Supplemental Figure 1G uncropped WB

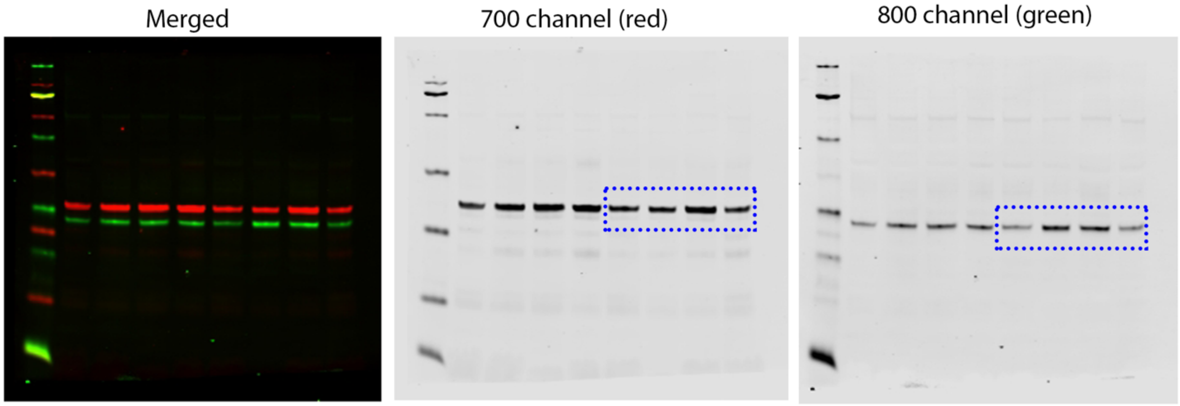

Supplemental Figure 3 uncropped WB

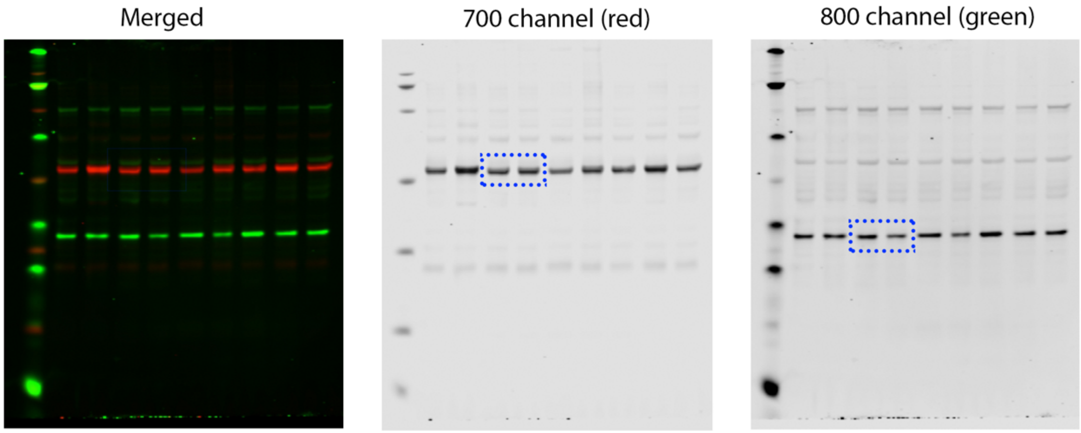

Supplemental Figure 5 uncropped WB

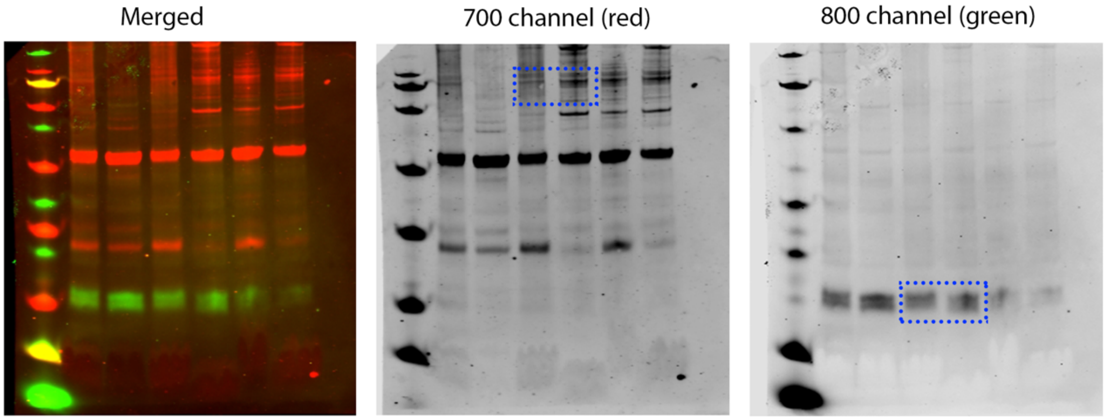

Supplemental Figure 11. Supplemental Figure 1G uncropped Western blot for GAPDH (700 channel, red) and CYB5R3 (800 channel, green). Blue hashed box shows cropped area. Supplemental Figure 3 uncropped Western blot for tubulin (700 channel, red) and CYB5R3 (800 channel, green). Blue hashed box shows cropped area. Supplemental Figure 5 uncropped

Western blot for tubulin (700 channel, red) and LC3 I/II (800 channel, green). Blue hashed box shows cropped area.

Supplemental Figure 7B uncropped WB

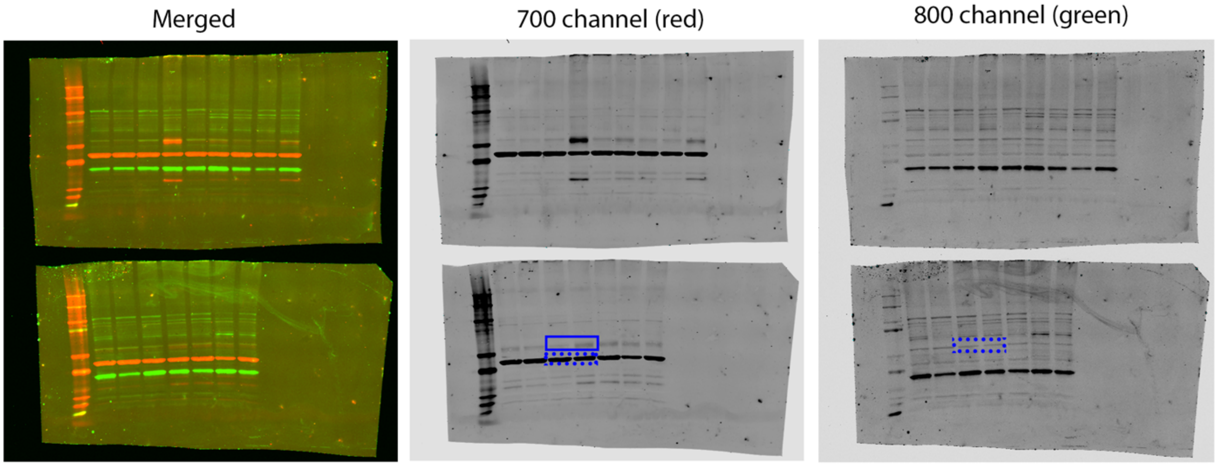

Supplemental Figure 7C uncropped WB

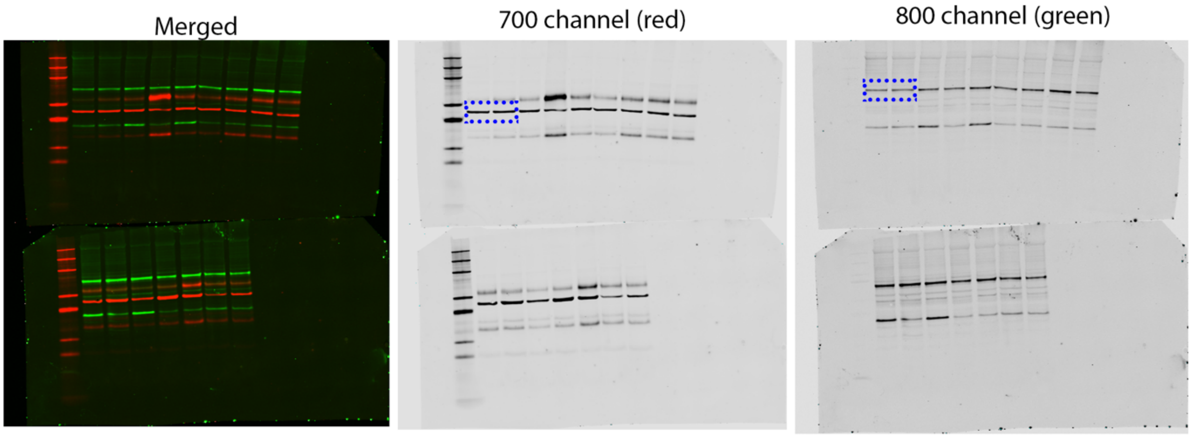

Supplemental Figure 12. Supplemental Figure 7B uncropped Western blot for β-actin and VASP (700 channel, red) and total p-VASP (800 channel, green). Blue solid box shows cropped area for VASP and blue hashed box shows cropped area for β-actin and p-VASP. Supplemental figure 7C uncropped Western blot for β-actin (700 channel, red) and sGC (800 channel, green). Blue hashed box shows cropped area.
